# Strong Association between Proanthocyanidins and Polysaccharides in the Cell Walls of Western Redcedar Bark

**DOI:** 10.1021/acs.biomac.5c00271

**Published:** 2025-05-06

**Authors:** Gio Ferson M. Bautista, Oliver Musl, Michael L. A. E. Easson, Lars H. Kruse, Harley Gordon, Markus Bacher, Ivan Sumerskii, Aude A. Watrelot, Jörg Bohlmann, Antje Potthast, Thomas Rosenau, Orlando J. Rojas

**Affiliations:** † Department of Chemistry, 8166 The University of British Columbia, 2036 Main Mall, Vancouver, British Columbia V6T 1Z1, Canada; ‡ Bioproducts Institute, The University of British Columbia, 2385 East Mall, Vancouver, British Columbia V6T 1Z4, Canada; § Department of Natural Sciences and Sustainable Resources, Institute of Chemistry of Renewable Resources, University of Natural Resources and Life Science (BOKU), Tulln, Vienna A-3430, Austria; ∥ Michael Smith Laboratories, The University of British Columbia, Vancouver V6T 1Z4, British Columbia, Canada; ⊥ Faculty of Land and Food Systems, The University of British Columbia, 2357 Main Mall, Vancouver, British Columbia V6T 1Z4, Canada; # Core Facility “Analysis of Lignocellulosics” (ALICE), University of Natural Resources and Life Sciences, Tulln, Vienna A-3430, Austria; ¶ Department of Food Science and Human Nutrition, 1177 Iowa State University, 536 Farm House Lane, Ames, Iowa 50011, United States; ∇ Department of Botany, The University of British Columbia, 6270 University Blvd., Vancouver, British Columbia V6T 1Z4, Canada; ○ Department of Forest and Conservation Sciences, The University of British Columbia, 2424 Main Mall, Vancouver, British Columbia V6T 1Z4, Canada; ⧫ Department of Chemical and Biological Engineering, University of British Columbia, 2360 East Mall, Vancouver, British Columbia V6T 1Z3, Canada; †† Department of Wood Science, The University of British Columbia, 2424 Main Mall #2900, Vancouver, British Columbia V6T 1Z1, Canada; ‡‡ Department of Bioproducts and Biosystems, Vuorimiehentie 1, Aalto University, Espoo FI-00076, Finland

## Abstract

The co-occurrence of polysaccharides and proanthocyanidins in the aqueous extracts of western redcedar (*Thuja plicata* Donn; WRC) bark limits their commercial utilization. To better understand their association, proanthocyanidins and polysaccharides were extracted with cold water (3.4% w/w bark) and isolated as an alcohol-insoluble residue (AIR, 1.0% w/w bark). The polysaccharide content (∼30% w/w AIR) was analyzed by acidic and enzymatic depolymerization, revealing the presence of pectins, xyloglucans, and xylans. NMR spectroscopy identified features, such as acetylation and methyl esterification. Thiolysis followed by HPLC-DAD revealed that proanthocyanidins (1.46% w/w AIR) exhibit a mean degree of polymerization of 5.3, a *cis*/*trans* ratio of 0.40, and a procyanidin/prodelphinidin ratio of 3.90. This study provides a detailed structural characterization of proanthocyanidins and polysaccharides in the AIR of WRC bark. The findings highlight their strong association, which may contribute to distinctive properties that warrant further exploration, particularly in efforts to valorize bark residues.

## Graphical abstract

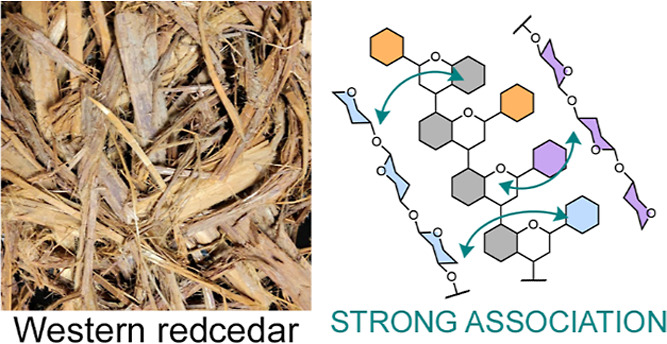

## 1 Introduction

Western redcedar (*Thuja plicata* Donn; WRC) is an economically and culturally important tree species in British Columbia and the greater Pacific Northwest. Bark residues account for a significant portion of the byproducts and are sold for the extraction of specialty chemicals. The bark of WRC is composed primarily of lignin (∼35–45%), cellulose (∼15–35%), hemicelluloses (∼20–30%), and various extractable components (∼10%).^1,2^ Organic solvent extracts from cedar bark contain a diverse array of compounds, often present in small quantities, which include monomeric polyphenols (e.g., flavanonols, hydroxycinnamic acids, flavanols), phenolic acids, aldehydes,^3^ fats and waxes,^4^ xanthoperol,^5^ isopimarinol and norditerpene alcohols,^6^ deoxypicropodophyllin,^7^ and phlobaphenes.^8^ In 1992, Hergert^9^ provided a detailed chemical description of water-extractable proanthocyanidins from the inner and outer bark of industrially utilized conifers, including WRC.

Despite their promising potential, the commercial utilization of WRC proanthocyanidins remains limited due to their low yield (∼4–7%) and low purity due to the co-occurrence of unknown polysaccharides.^2^ However, increasing interest in proanthocyanidins for applications in nutraceuticals, functional foods and feeds, leather tanning, wine production, wood adhesives, and polysaccharide-based products has driven research into novel uses for these compounds. This study aims to investigate the chemical nature of the aqueous extract of WRC bark to better understand its components and explore its potential for economic valorization.

Proanthocyanidins (syn. condensed tannins) are oligomeric and polymeric flavan-3-ols found in many plant tissues and organs, including seeds, leaves, bark, wood, and roots.^10^ Most common in nature are procyanidins and prodelphinidins, which are composed of (epi)­catechin and (epi)­gallocatechin, respectively. The flavan-3-ol building blocks polymerize within tannosomes in chloroplasts, after which they are transported in membrane-bound structures to vacuoles for storage.^11^ In conifer bark fibers, polyphenolic parenchyma cells store polyphenols, including proanthocyanidins, which contribute to plant defense mechanisms.^12^ Proanthocyanidins possess a rich inventory of biological activities. They exhibit pro-oxidant activity,^13^ bind proteins, inhibit digestive enzymes,^14^ and damage the digestive tract of pests. Proanthocyanidins demonstrate antimicrobial activity by binding hydrolytic enzymes (e.g., cellulases, pectinases, peroxidases) and chelating metal ions (e.g., Fe^3+^/Fe^2+^) essential for microbial nutrition.^14^ Notably, proanthocyanidins are also localized within plant cell walls,^15^ where their interactions with cellulose, hemicelluloses, and pectins impart unique properties critical for plant defense.

The plant cell wall is a complex composite of cellulose, hemicelluloses, pectins, glycoproteins, and various polyaromatic structures.^16^ This intricate matrix provides an environment for complex interactions with proanthocyanidins, which may influence their functional properties. Recent advances in proanthocyanidin research have highlighted key structural features important for polysaccharide association, such as the degree of polymerization (DP), galloylation, and the stereochemistry of C2 and C3 in the benzopyran rings.^17^ Similarly, the structural and conformational organization of polysaccharides dictates their affinity and degree of interaction with proanthocyanidins, as demonstrated in studies on apple cell wall material and purified polysaccharides.^18–20^ While the association per se between proanthocyanidins and polysaccharides in bark extracts is acknowledged, it remains poorly characterized due to the structural complexity of both components and the heterogeneity and variability of their mixtures. This study seeks to address these challenges by investigating the structure and composition of proanthocyanidins and polysaccharides for a particular example, the aqueous extract of WRC bark. By elucidating the nature of their association, we aim to provide insights that will inform future efforts to valorize this abundant yet still underutilized raw material.

## 2 Materials and Methods

### 2.1 Chemicals and Materials

The stem bark of WRC (*T. plicata* Donn) was provided by the Teal Jones Group sawmill (Surrey, BC, V4N 4M8, Canada). The bark originated from trees felled in Terrace, BC, and transported via the Fraser River before being debarked at the Surrey sawmill. The air-dried bark was ground using a pilot-scale rotor mill, passed through a 10-mesh sieve, and stored in tightly sealed plastic bags at ambient conditions until use. At the time of extraction, the air-dried bark exhibited a moisture content of 9.28 ± 0.18%, as determined by a moisture analyzer (HC103, Mettler Toledo, Greifensee, Switzerland).

Flavan-3-ol standards, including (−)-epicatechin, (+)-catechin hydrate, (−)-epigallocatechin, and (−)-gallocatechin, were purchased from Sigma-Aldrich (St. Louis, MO, USA). Standards for procyanidins B2 and B3 were obtained from Extrasynthese (Lyon, France). Monosaccharides, disaccharides, polysaccharides, and other carbohydrate standards, including l-arabinose, d-galactose, d-glucose, d-xylose, d-mannose, l-rhamnose, l-fucose, d-glucuronic acid, d-galacturonic acid, d-sorbitol, cellobiose, native starch (wheat), Avicel PH-101, pectin (citrus peel), and xylan (birchwood), were obtained from Sigma-Aldrich (St. Louis, MO, USA). Additional standards included 4-*O*-methyl-d-glucuronic acid from Biosynth (Staad, Switzerland), isoprimeverose from AABlocks (San Diego, CA, USA), and β-glucan (barley, high viscosity) from Megazyme (Bray, Ireland). Xylobiose and tamarind gum were purchased from TCI (Tokyo, Japan), while sainfoin leaves were obtained from StableFeed (Kasson, MN, USA), and quaking aspen leaves were donated by the UBC Botanical Garden (Vancouver, BC, Canada).

Enzymes used in this study included Termamyl 2X (α-amylase from *Bacillus licheniformis*, 20.2 mg protein/mL, 826 units/mg protein), amyloglucosidase (from *Aspergillus niger*, 142 units/mg solids), and Driselase (from *Basidiomycetes* sp., 21% w/w protein, 551 units cellulase/g solid, 433 units laminarinase/g solid, 6 units xylanase/g solid), all purchased from Sigma-Aldrich (St. Louis, MO, USA).

### 2.2 Preparation of Alcohol-Insoluble Residue from Bark

To extract the bark, 10 L of Milli-Q water was added to 1.5 kg of ground, air-dried bark. The bark suspension had an initial pH of 8.0, which naturally dropped to a range of 3.5–4.0 during the extraction process. The mixture was stirred overnight at room temperature using an overhead stirrer (Eurostar 20 digital, IKA, NC, USA) in a covered container lined with aluminum foil. After stirring, the supernatant was separated from the bark using a sieve and centrifuged at 20,000 × *g* at 20 °C for 30 min in a refrigerated centrifuge (Sorvall Savant Lynx 6000, ThermoScientific). The resulting supernatant was vacuum-filtered through Whatman qualitative filter paper (grade 1) and then lyophilized on a 50 L pilot-scale freeze-dryer (VirTis VirTual, SP Industries, PA, USA). The lyophilized product, a brown powder (46.9 g, 3.35% (w/w) dried bark), was stored in an amber bottle at 4 °C.

Precautions were taken throughout the study to minimize chemical alterations of the samples, avoiding high temperatures, strong acids or bases, oxidation, or biochemical degradation (e.g., fermentation or enzymatic action). Cold water (∼20 °C) was used as a mild extractant^21^ to prevent the cleavage of labile bonds and to facilitate the detection of associations between cell wall polymers. The use of sodium sulfite or metabisulfite was avoided to prevent potential degradation of proanthocyanidin-polysaccharide bonds and the depolymerization of proanthocyanidins into flavan-3-ol sulfonates.^22^

The preparation of the alcohol-insoluble residue (AIR) was adapted from the method described by Hergert.^3^ The lyophilized aqueous bark extract was first washed with pure methanol, followed by 75% (v/v) acetone in water. For the methanol washing, the dried aqueous extract was redispersed in a minimal amount of water and then diluted with 20 volumes of methanol (final methanol-to-water ratio of 20:1 v/v). The methanol-insoluble residue was recovered by centrifugation. This residue was subsequently redispersed in a minimal amount of water and diluted with acetone to achieve 75% (v/v) acetone. Centrifugation yielded an insoluble residue, which was then lyophilized into a brown powder designated as AIR (14.0 g, 1.00% w/w dried bark). The AIR was stored at 4 °C, and aliquots were taken for further analysis. A summary of the procedure is provided in Scheme 1.

**Scheme 1:**
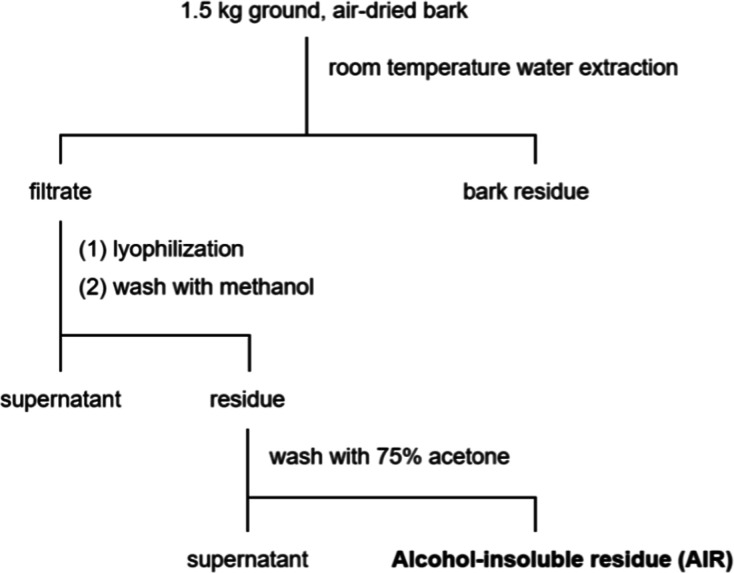
Preparation of WRC Bark AIR

### 2.3 Determination of Monosaccharide Composition by Total Hydrolysis and HPAEC–PAD

The monosaccharide composition of AIR was determined according to the method described by Shevchenko et al.^23^ Briefly, 30 mg of lyophilized, ground AIR (40 mesh) was weighed into a footed wide-mouth borosilicate beaker (Klason cup). Hydrolysis was performed in two stages: first, 3 mL of 72% (w/w) H_2_SO_4_ was added, and the sample was stirred with a glass rod for 2 h. For the second stage, the mixture was diluted with Milli-Q water to a total volume of 115 mL and autoclaved at 120 °C and 14.9 psi for 1 h. The hydrolysate was filtered through a preweighed Gooch filter crucible with a sintered disc. The filtrate was analyzed for monosaccharides using high-performance anion-exchange chromatography with pulsed amperometric detection (HPAEC-PAD) (details in Supplementary Methods). Peaks in the chromatograms were integrated using MestReNova V15.0.1 software (A Coruña, Spain) and quantified using calibration curves established using monosaccharide analytical standards including l-arabinose, d-galactose, d-glucose, d-xylose, d-mannose, l-rhamnose, l-fucose, and d-sorbitol (internal standard).

### 2.4 Determination of Monosaccharide Composition by Acidic Methanolysis and GC–MS

For acidic methanolysis, 20 mg of lyophilized AIR was dispersed in 4 mL of 2 M HCl in anhydrous methanol and incubated at 100 °C for 3 h. After lyophilization, the hydrolysate was derivatized by trimethylsilylation with a mixture of 4-(dimethylamino)­pyridine and *N*,*O*-bis­(trimethylsilyl)­trifluoroacetamide (BSTFA) containing 10% trimethylchlorosilane (TMS-Cl). The derivatized sample was dissolved in 600 μL of ethyl acetate and injected (0.2 μL) into a gas chromatograph–mass spectrometer for analysis (details in Supplementary Methods), according to the procedure by Liftinger et al.^24^ Peak identification was done by comparison with silylated analytical standards of l-arabinose, d-galactose, d-glucose, d-xylose, d-mannose, l-rhamnose, l-fucose, d-glucuronic acid, d-galacturonic acid, 4-*O*-methyl-d-glucuronic acid, and myo-inositol. A mixture of ^13^C-labeled products from alkaline glucose degradation was used as internal standard for quantification.^24^

### 2.5 Determination of Monosaccharides and Disaccharides by Driselase Digestion and HPAEC–PAD

Driselase digestion was performed as described by Fry^25^ and Al Hinai et al.^26^ A 0.2% (w/w) Driselase enzyme solution was prepared in 100 mM sodium acetate buffer (pH 5) containing 0.01% (w/v) NaN_3_, followed by centrifugation at 21,000*g* for 30 min at 4 °C to remove insoluble residues. 100 mg of AIR was dispersed in 5 mL of the enzyme solution and incubated at 37 °C for 3 d while shaking. Ethanol was added to an 80% (v/v) final concentration to precipitate the enzyme, which was then removed by centrifugation at 3000 x *g* for 10 min at 15 °C. The supernatant was dried under vacuum, reconstituted in Milli-Q water, and spiked with D-sorbitol (internal standard). Samples were analyzed by HPAEC–PAD for monosaccharides, uronic acids, and disaccharides (details in the Supplementary Methods). Analytical standards and reference samples were used for peak identification and quantification. Peaks in the chromatograms were integrated using MestReNova V15.0.1 software (A Corua, Spain) and quantified using calibration curves established using monosaccharide analytical standards including l-arabinose, d-galactose, d-glucose, d-xylose, d-mannose, l-rhamnose, l-fucose, d-glucuronic acid, 4-*O*-methyl-d-glucuronic acid, d-galacturonic acid, and d-sorbitol (internal standard).

### 2.6 Determination of Proanthocyanidin Monomer Subunits by Thiolysis and HPLC-DAD-MS

The monomeric subunits of proanthocyanidins in AIR were determined by thiolysis according to the method by Callemien et al.^27^ A 10 mg portion of AIR was reacted with 50 μL of 3.3% (v/v) concentrated HCl in methanol and 100 μL of 5% (v/v) benzylthiol in methanol. The reaction mixture was vortexed, heated to 40 °C for 30 min, and then incubated at room temperature for 48 h with orbital shaking. After centrifugation (21,000 × *g*, 5 min), the supernatant was analyzed by high-performance liquid chromatography with diode array detection (HPLC–DAD) and mass spectrometry. Proanthocyanidin terminal and extension units were quantified at 280 nm, and structural properties, such as mean degree of polymerization, procyanidin/prodelphinidin ratio, and *cis*/*trans* ratio, were calculated based on calibration curves from flavan-3-ol analytical standards^28^ using eqs 1–3. A control sample dispersed in methanol was also processed and injected to correct for the quantities of terminal units in the thiolyzed sample. The epimerization rates of analytical standards of epicatechin (67% mol) and catechin (29% mol) were determined under the thiolysis conditions to correct for the quantities of epicatechin and catechin in WRC bark AIR, respectively. The assignments of epimeric pairs (3,4-*trans* vs 3,4-*cis*) of adducts are based on the relative elution orders reported by Ramsay et al.^29^ Peaks in the chromatograms were integrated using MestReNova V15.0.1 software (La Corua, Spain) and quantified using calibration curves established using flavan-3-ol analytical standards including (−)-epicatechin, (+)-catechin hydrate, (−)-epigallocatechin, and (−)-gallocatechin.

1 $$mDP=\frac{extension\;units+terminal\;units}{terminal\;units}=\frac{n_{1}+n_{2}+...+n_{10}-\left(n_{1c}+n_{2c}+n_{3c}+n_{4c}\right)}{n_{1}+n_{2}+n_{3}+n_{4}-\left(n_{1c}+n_{2c}+n_{3c}+n_{4c}\right)}$$

2 $$cis:trans\;ratio=\frac{2,3‐cis\;units}{2,3‐trans\;units}=\frac{n_{2}+n_{4}+n_{7}+n_{10}-\left(n_{2c}+n_{4c}\right)}{n_{1}+n_{3}+n_{5}+n_{6}+n_{8}+n_{9}-\left(n_{1c}+n_{3c}\right)}$$

3 $$PC:PD\;ratio=\frac{procyanidin\;units}{prodelphinidin\;units}=\frac{n_{3}+n_{4}+n_{8}+n_{9}+n_{10}-\left(n_{3c}+n_{4c}\right)}{n_{1}+n_{2}+n_{5}+n_{6}+n_{7}-\left(n_{1c}+n_{2c}\right)}$$

where *n*_*x*_ refers to the moles of compound per g of sample after thiolysis and the numerical subscript *x* refers to compound designations as listed in Table 2; and *n*_*x*c_ refers to the moles of compound per g of sample in the control.

**Table 1:** Monosaccharide Content of Dried WRC Bark, WRC Bark after Extraction, and AIR

	mass of monosaccharide ± SD (*n* = 3)		
material	WRC bark (% w/w in unextracted dried bark)	WRC bark after extraction (% w/w in unextracted dried bark)	AIR (%w/w in AIR)
total hydrolysis in H_2_SO_4_ ^a^	43 ± 4	43 ± 7	18 ± 2
Glu	30 ± 3	31 ± 5	9 ± 1
Ara	1.14 ± 0.08	0.9 ± 0.2	1.9 ± 0.4
Gal	1.2 ± 0.1	1.0 ± 0.3	2.1 ± 0.5
Xyl	4.1 ± 0.5	3.9 ± 0.6	2.28 ± 0.07
Man	6.2 ± 0.5	6.5 ± 0.9	2.7 ± 0.1
Rha	0 (not detected)	0 (not detected)	0 (not detected)
Fuc	1.03 ± 0.03	0.7 ± 0.2	1.47 ± 0.07
acid-insoluble residue after total hydrolysis^b^	62 ± 8	51 ± 4	90 ± 10
acidic methanolysis^c^	14.5 ± 0.5	13.7 ± 0.4	21.9 ± 0.6
Glu	1.37 ± 0.06	1.30 ± 0.04	2.9 ± 0.1
Ara	1.31 ± 0.06	1.15 ± 0.05	2.4 ± 0.1
Gal	1.16 ± 0.09	1.07 ± 0.05	2.5 ± 0.8
Xyl	2.7 ± 0.01	2.59 ± 0.09	1.72 ± 0.07
Man	3.5 ± 0.3	3.5 ± 0.2	1.85 ± 0.06
Rha	0.34 ± 0.04	0.29 ± 0.03	1.33 ± 0.04
Fuc	0.21 ± 0.04	0.19 ± 0.02	0.40 ± 0.02
GalA	2.3 ± 0.3	2.0 ± 0.2	7.8 ± 0.3
4-*O*-MeGlcA	1.64 ± 0.09	1.6 ± 0.1	0.59 ± 0.02
GlcA	0 (not detected)	0 (not detected)	0.41 ± 0.02
Driselase digestion (% w/w)^a^	n/a^d^	n/a^d^	11.3 ± 0.9
Glu			1.3 ± 0.3
-Glu from isoprimeverose			0.34 ± 0.03
-Glu as a monosaccharide			1.0 ± 0.3
Ara			0.80 ± 0.06
Gal			0.84 ± 0.06
Xyl			1.06 ± 0.05
-Xyl from xylobiose			0.55 ± 0.04
-Xyl from isoprimeverose			0.28 ± 0.02
-Xyl as a monosaccharide			0.228 ± 0.009
Man			0.238 ± 0.001
Rha			0.338 ± 0.008
Fuc			0.56 ± 0.07
GalA			6.1 ± 0.9
4-*O*-MeGlcA			0 (not detected)
GlcA			0 (not detected)

a Quantified by HPAEC–PAD.

b Dry sample basis.

c Quantified by GC–MS.

d Not analyzed. Abbreviations are defined as follows: Glu—glucose; Ara—arabinose; Gal—galactose; Xyl—xylose; Man—mannose; Rha—rhamnose; Fuc—fucose; GalA—galacturonic acid; 4-*O*-MeGlcA—4-*O*-methylglucuronic acid; GlcA—glucuronic acid.

**Table 2:** Retention Time and Mass Spectra (MS) Information for Proanthocyanidin Terminal and Extension Units after Thiolysis

peak no.	compound	retention time, min	MŜn Precursor and fragment ions (−ve), *m*/*z*	amount ± SD (% w/w)^a^ (*n* = 3)
1	(+)-gallocatechin	2.0	419.0 → 304.9, 288.9	0.054 ± 0.004
2	(−)-epigallocatechin	2.5	419.0 → 304.9, 288.9	0.008 ± 0.001
3	(+)-catechin	3.2	403.0, 289.0	0.121 ± 0.005
4	(−)-epicatechin	4.5	403.0, 289.0	0.095 ± 0.005
5	3,4-*trans*-gallocatechin benzyl thioether	10.4	541.2 → 427.0, 302.9	0.072 ± 0.008
6	3,4-*cis*-gallocatechin benzyl thioether	10.7	541.2 → 481.1, 427.0, 303.1, 177.0	0.045 ± 0.002
7	3,4-*trans*-epigallocatechin benzyl thioether	11.2	541.2 → 416.9, 302.9	0.139 ± 0.05
8	3,4-*trans*-catechin benzyl thioether	13.0	525.2 → 400.9, 286.7	0.119 ± 0.002
9	3,4-*cis*-catechin benzyl thioether	14.2	525.2 → 410.9 → 286.9, 243.0, 125.0	0.70 ± 0.02
10	3,4-*trans*-epicatechin benzyl thioether	15.6	525.3 → 401.1, 287.0	0.110 ± 0.003
total proanthocyanidin yield (% w/w)^a^	1.46 ± 0.04			
number-average DP	5.3 ± 0.2			
number-average MW, kDa	1.32 ± 0.05			
PC/PD ratio	3.90 ± 0.83			
*cis*/*trans* ratio	0.40 ± 0.04			

a Based on the dry weight of AIR.

### 2.7 Structural Characterization by Nuclear Magnetic Resonance Spectroscopy

NMR spectra were recorded in DMSO-*d*_6_ using a Bruker AVANCE II 400 or AVANCE III HD 400 (400.13 MHz for ^1^H, 100.63 MHz for ^13^C, and ^31^P at 161.98 MHz). The instruments were equipped with either a broadband probe head (BBFO) or a cryoprobe (Prodigy) with z-gradients. Chemical shifts were referenced to residual solvent signals (2.49 ppm for ^1^H, 39.6 ppm for ^13^C).

#### 2.7.1 Polymer Species Characterization by Diffusion-Edited ^1^H NMR

A 50 mg portion of AIR was dissolved in a 4:1 (w/w) mixture of DMSO-*d*_6_ and tetra-*n*-butylphosphonium acetate ([P_4444_]­[OAc]). Parameters were adopted from Fliri et al.^30^

#### 2.7.2 Polysaccharide Structure by ^1^H–^13^C HSQC NMR

Samples (50 mg) were dissolved in 0.6 mL of DMSO-*d*_6_ or a 4:1 (w/w) mixture of DMSO-*d*_6_ and [P_4444_]­[OAc].^30^ HSQC spectra were acquired in edited mode with a relaxation delay of 0.5 s using an adiabatic pulse for the inversion of ^13^C and the GARP-sequence for broadband ^13^C-decoupling, optimized for ^1^ *J*_C,H_ = 145 Hz.

#### 2.7.3 Quantification of Hydroxyl Groups by Phosphitylation and ^31^P NMR

Hydroxy group contents (aliphatic hydroxyls, aromatic hydroxyls, and carboxylic acids) were determined by inverse gated ^1^H-decoupled ^31^P NMR spectroscopy. Thirty mg of AIR was dissolved in a mixture of *N*,*N*-dimethylformamide (DMF, 600 μL) and pyridine (Pyr, anhydrous, nondeuterated, 100 μL). Internal standard (4 mg of *N*-hydroxy-5-norbornene-2,3-dicarboxylic acid imide) and 0.5 mg of NMR relaxation agent (chromium­(III) acetylacetonate, (Cr­(acac)_3_), were added along with the solvent mixture. For phosphitylation, 150 μL of 2-chloro-4,4,5,5-tetramethyl-1,3,2-dioxaphospholane (TMDP-Cl) was used. 50 μL of chloroform-*d* was added for solvent locking. Spectra were calibrated using the signal of the hydrolysis byproduct formed through the reaction of the derivatization reagent TMDP with water (δ = 132.2 ppm). Sample peaks were assigned and integrated based on regions reported by Meng et al.^31^ using Topspin 4.3.0 (MA, USA).

## 3 Results and Discussion

### 3.1 Extraction and Isolation of WRC Bark AIR

The WRC (*T. plicata*) bark used in this study was obtained from river-run logs debarked at a sawmill. The bark was delivered to the laboratory within a week, air-dried, and ground for subsequent extraction. Water extraction was selected as a straightforward method to recover water-extractable polysaccharides and phenolic polymers in a single step, as previously reported by Kurth.^2^ This technique is technologically simple and can be readily implemented at sawmills or other wood-processing facilities.^32^ Importantly, no additives or elevated temperatures were used in this study to avoid chemical modifications in the extract, which are known to occur under such conditions (as noted in Section 2.2). The inclusion of additives or cosolvents would complicate the downstream utilization of residual bark, necessitating chemical or solvent recovery before it can be repurposed for applications such as mulch, soil cover, or as a biofuel for energy generation.^32^ Nevertheless, water consumption and the energy required for drying remain key sustainability concerns, as highlighted in studies on aqueous extraction of spruce bark.^33^

Aqueous extraction yielded 3.4% (w/w) lyophilized material based on the oven-dried bark. The dried extract was subsequently resuspended in water and purified using methanol and aqueous acetone to remove fatty substances, weakly bound polyphenols, lignin, and low-molecular-weight lignin-carbohydrate complexes.^34^ Both methanol and aqueous acetone washings were found to contain proanthocyanidins, as indicated by the appearance of red pigments following acidic butanol hydrolysis. This observation suggests that a portion of the proanthocyanidins can be selectively separated from the associated polysaccharides using neutral organic solvents, yielding a purified fraction with the potential for high-value applications in the food and nutraceutical industries. Such solvent-based purification is commonly used in commercial extraction facilities to refine tannin-rich extracts.^32^ The purification also yielded an AIR, accounting for approximately 1% (w/w) of the dried bark. This WRC bark AIR was isolated to obtain polysaccharide fractions with minimal contamination from loosely bound compounds. Elemental analysis of AIR revealed a composition of 50.2% carbon, 5.2% hydrogen, and 0.9% nitrogen. The protein content was estimated to be 5.6% (w/w) based on a nitrogen-to-protein conversion factor of 6.25.

The following sections will detail the analysis of the polysaccharide structures within the AIR, the structural characterization of the proanthocyanidins, and a discussion of potential noncovalent interactions between these components. The results are supported by solubility and dispersibility tests conducted during the study.

### 3.2 Polysaccharide Analysis

#### 3.2.1 Polysaccharide Composition of WRC Bark

The polysaccharide composition of the materials obtained in this study is summarized in Table 1. We have found it necessary to use both total hydrolysis and acidic methanolysis to obtain maximum information on the changes in the polysaccharide composition of the bark after extraction. Acidic methanolysis was employed to analyze hemicelluloses and pectins because some of their structural components are susceptible to degradation during total hydrolysis. Total hydrolysis with H_2_SO_4_ revealed that glucans are the primary polysaccharide fraction in WRC bark. Cellulose is likely the main glucan present, with additional hemicellulosic glucans inferred from the results of acidic methanolysis. Other neutral monosaccharides, such as xylose (Xyl) and arabinose (Ara), were also detected. These are presumed to originate from arabino-4-*O*-methylglucuronoxylan in WRC wood.^34^ This is further supported by the detection of glucuronic acid (GlcA) and 4-*O*-methylglucuronic acid (4-*O*-MeGlcA) upon acidic methanolysis.

Mannans, including glucomannans and galactoglucomannans, are known to occur in WRC wood^34,35^ and are likely present in the bark as well. However, the apparent enrichment of mannose (Man) in the bark after extraction, observed through total hydrolysis in H_2_SO_4_, indicates that mannose-rich polysaccharides, possibly connected to their degree of acetylation, exhibit minimal extractability in cold water. This observation aligns with previous reports concerning WRC wood that mannose-rich polysaccharides are rather best extracted with sodium hydroxide.^36^

The presence of galacturonic acid (GalA) confirms that pectins are also a component of WRC bark, constituting more than 2.3% (w/w). However, both total hydrolysis and acidic methanolysis results indicate that only a small fraction (0.4% w/w) of the total polysaccharides, likely pectins, were extracted using cold water. Nevertheless, polysaccharides represent a significant portion (∼30% w/w) of the AIR, highlighting their importance in the composition of the WRC bark extract.

#### 3.2.2 Acid-Insoluble Residues after Hydrolysis

The high amount of acid-insoluble residue (51–62%) observed after total hydrolysis in H_2_SO_4_ of the bark reflects the lignin content, as well as contributions from polymeric polyphenols, such as proanthocyanidins and phlobaphenes,^37^ protein residues, and lipid substances, such as suberin.^38^ The removal of water-soluble components from the bark resulted in an extracted bark sample with significantly less acid-insoluble residue after total hydrolysis. Conversely, AIR exhibited an unusually high acid-insoluble residue content. This suggests that the AIR fraction contributes significantly to the acid-insoluble residues in the bark samples.

#### 3.2.3 Polysaccharide Analysis of AIR by Hydrolysis and Enzymatic Digestion

##### 3.2.3.1 Correcting for Starch Content

WRC bark contains considerable amounts of water-extractable starch, as starch is commonly present in phloem tissue. Since starch is insoluble in methanol or aqueous acetone, it coprecipitated in AIR, contributing 1.9 g of starch per 100 g of AIR (measured as monomeric glucose). To accurately reflect the composition of cell wall polysaccharides, glucose values obtained from total hydrolysis in H_2_SO_4_ and acidic methanolysis of AIR were corrected for the starch content (as shown in Figure 1), as starch is not considered a cell wall component. The molar and weight ratios of sugars in AIR are thus assumed to represent the water-extractable components of WRC bark cell wall polysaccharides.

**Figure 1:**
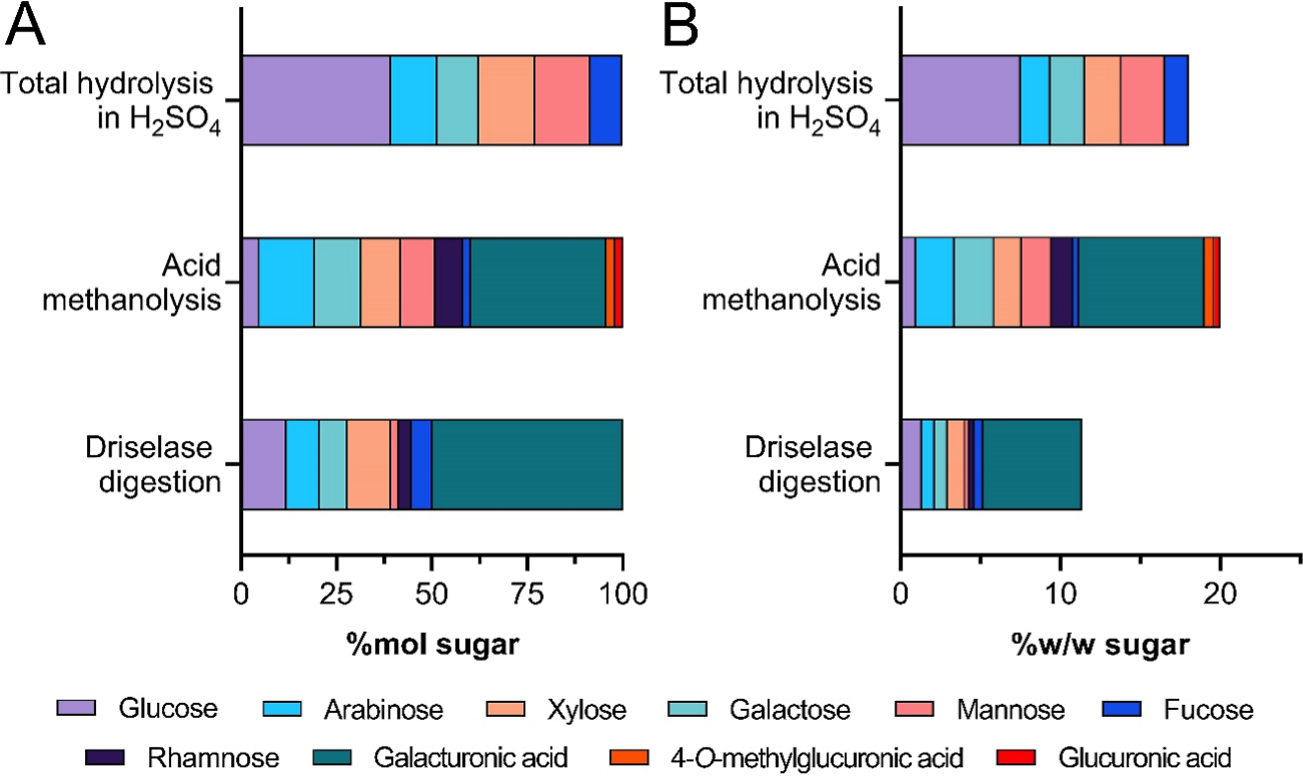
Molar percentages (A) and weight percentages (B) of monosaccharides in AIR using different polysaccharide depolymerization methods. Monosaccharides from total hydrolysis in H_2_SO_4_ and Driselase digestion were quantified by HPAEC–PAD, while acidic methanolysis products were quantified by GC–MS. The glucose yield in total hydrolysis in H_2_SO_4_ and acidic methanolysis was corrected for the starch content.

##### 3.2.3.2 Total Hydrolysis in H_2_SO_4_

Total hydrolysis of AIR with H_2_SO_4_ produced monosaccharides in the molar abundance order of Glu ≫ Man ∼ Xyl ∼ Ara ∼ Gal > Fuc. The predominance of glucose suggests the presence of a glucan backbone. The measured contents of mannose (Man), xylose (Xyl), arabinose (Ara), and galactose (Gal) indicate the presence of hemicelluloses, such as (galacto)­glucomannans, (arabino)­xylans, and arabinogalactans. Since cellulose shows no water extractability, the glucose-containing polysaccharides extracted into AIR are of noncellulosic origin.

##### 3.2.3.3 Acidic Methanolysis

Acidic methanolysis was employed to analyze hemicelluloses and pectins because some of their structural components are susceptible to degradation during total hydrolysis. In particular, uronic acids tend to undergo decarboxylation and follow-up-degradation under acidic hydrolysis conditions (H_2_SO_4_) while being stable upon acidic methanolysis. Acidic methanolysis of AIR produced monosaccharides, including uronic acids, in the molar abundance order: GalA ≫ Ara > Gal > Xyl > Man > Rha > Glu >4-*O*-MeGlcA ∼ Fuc > GlcA. The lower glucose yield compared to total hydrolysis suggests that most glucose originates from a more recalcitrant polysaccharide. The relative abundances of GalA and rhamnose (Rha) indicate that pectins form a significant fraction of the AIR. Additionally, the broad and evenly distributed sugar composition suggests the presence of several different hemicelluloses.

##### 3.2.3.4 Enzymatic Hydrolysis with Driselase

Enzymatic hydrolysis with Driselase provided further insight into the glycosidic linkages between the monosaccharide constituents. Driselase, a fungal enzyme mixture with both endo- and exoglycanase activities,^39^ is known to digest major plant cell wall polysaccharides, such as polygalacturonan, rhamnogalacturonan, xylan, cellulose, laminarin, galactan, arabinan, and mannan.^40,41^ Driselase is also tolerant of polyphenol cross-links, including ferulates, diferulates, and *p*-coumarates.^42^

Driselase digestion yielded only half the amount of sugars produced by total hydrolysis or acidic methanolysis. The monosaccharide composition of the Driselase digest followed the molar abundance order: GalA > Xyl ∼ Glu > Ara > Gal > Fuc > Rha > Man. This pattern aligns with the results from acidic hydrolysis and methanolysis, except for glucose and mannose. Glucose- and mannose-containing polysaccharides, such as xyloglucans, galactoglucomannans, and pectin-proteoglycans (e.g., arabinogalactan proteins), appear resistant to complete Driselase digestion. In particular, the pectin proteoglycans are known to resist enzymatic breakdown and may contain mannose residues.^43^

##### 3.2.3.5 Limitations and Insights from Monosaccharide Analyses of AIR

The monosaccharide analyses employed in this study provide only an approximate measure of polysaccharide quantities in AIR. This is partly due to the low recovery of sugars observed with the methods used and the significant proportion of recovered insoluble material (summarized in Supplementary Table S1). Acidic depolymerization techniques, such as acidic methanolysis and H_2_SO_4_ hydrolysis, are prone to different side reactions that can both deplete monosaccharide products and cause incomplete hydrolysis. Factors contributing to these limitations include physical barriers, such as high crystallinity, and chemical properties, such as the presence of polyuronic acid chains,^44^ alkyl residues, or cross-links.

Additionally, AIR contains nonpolysaccharide components, such as aliphatic extractives, proteins, and polyphenolic substances, which further complicate monosaccharide recovery. The large amount of insoluble material remaining after acidic hydrolysis suggests that either the AIR itself or the byproducts of side reactions are rather recalcitrant to hydrolysis. Polyphenols, such as proanthocyanidins and lignin, along with proteins, are known to interfere with acidic depolymerization methods.^37^

AIR showed differences in acid-insoluble residue content depending on the hydrolysis method, with 48% (w/w) by acidic methanolysis compared to 88% (w/w) by total hydrolysis with H_2_SO_4_ (Supplementary Table S1). This highlights the complex behavior of the WRC bark extract during acidic depolymerization under different solvent conditions and acid concentrations (2 M HCl in acidic methanolysis versus 12 M H_2_SO_4_). Such complexity may involve side reactions, as seen in similar studies. For instance, Harpham^45^ reported unusually high amounts of precipitated mass from spruce bark aqueous extracts after hydrolysis in 0.4 M HCl, which could not be fully explained. It is likely that acid hydrolysis in aqueous media induces complex condensation reactions influenced by the polyphenol content of the bark,^46^ which cause polysaccharide alkylation and cross-linking. Therefore, the polysaccharide content of AIR is likely to be underestimated by the applied hydrolysis methods.

Driselase digestion yielded a lower sugar recovery than acidic hydrolysis methods, likely due to the endogenous acetylation of the polysaccharides. The diffusion-edited ^1^H NMR spectra (Figure 2A) revealed a signal at 1.99 ppm, consistent with the possible acetylation of arabinoglucuronoxylans, xyloglucans, and pectins.^47^ These polysaccharides, suspected to be present in AIR (as will be discussed below), are known to exhibit acetylation, which can hinder enzymatic hydrolysis (such as by pectinases and xylanases). Other factors, such as strong associations or alkyl linkages between polysaccharide components and polyphenolic substances (proanthocyanidins and lignin), are also known to inhibit enzymatic action.^48^

**Figure 2:**
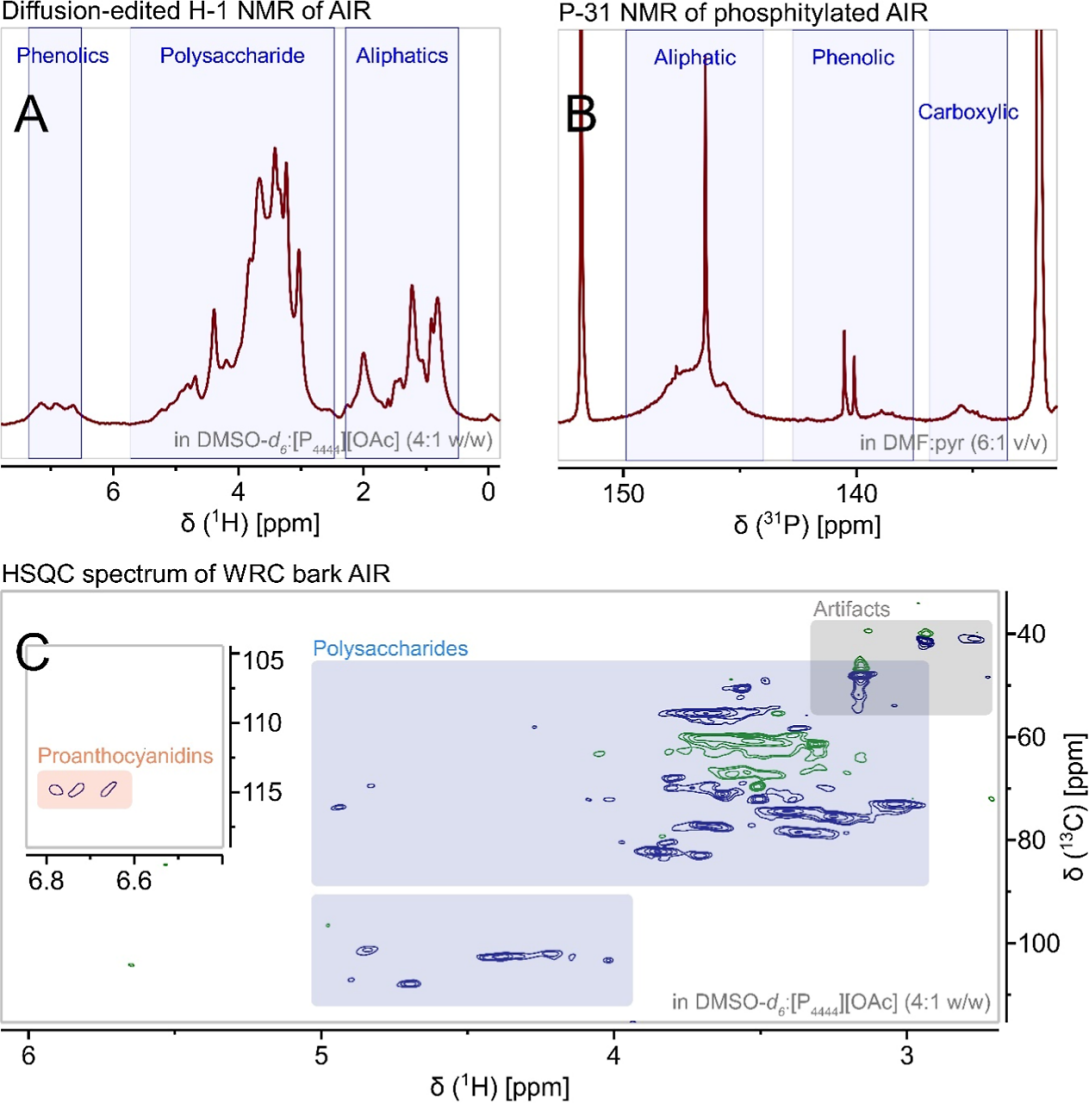
NMR spectra of AIR confirming the presence of both polysaccharides and proanthocyanidins. (A) ^1^H NMR, (B) ^31^P NMR, and (C) multiplicity-edited HSQC NMR (green CH_2_, blue CH/CH_3_).

Although the sugar recovery from enzymatic digestion was lower than that by acidic hydrolysis, the amount of insoluble material recovered was intermediate between those of acidic methanolysis and sulfuric acid hydrolysis. This suggests that some solubilized products were either not detectable or not identified. Examination of the HPAEC–PAD chromatogram for the Driselase digest of AIR (Supplementary Figure S1, inset) revealed peaks that may correspond to uronic acid oligosaccharides but were not identified.

##### 3.2.3.6 Insights from ^31^P NMR

Quantification of aliphatic hydroxyl groups by ^31^P NMR of phosphitylated AIR provided additional insights related to the depolymerization yields. Integration of the ^31^P NMR spectra (Figure 2B, Supplementary Table S2) revealed a low amount of free OH groups (1.7 mmol/g), consistent with the branched and acetylated nature of the polysaccharides in AIR.

#### 3.2.4 Reconstruction of AIR Polysaccharide Composition Using Degradative Methods

Polysaccharide analysis by degradative methods revealed a complex sugar composition derived from a range of different polysaccharides. To reconstruct the diverse polysaccharide composition of AIR, we estimated the relative contributions of certain monomeric units based on their measured abundances.

##### 3.2.4.1 Pectins

The relative content of GalA and Rha suggests a significant presence of homogalacturonan (HG) and rhamnogalacturonan I (RG-I) type pectins. Based on the GalA/Rha ratio of 4.9 observed in acidic methanolysis, HG chains appear to be roughly twice as abundant as RG-I chains. The uronic acids (0.5 mmol/g AIR based on acidic methanolysis) appear to be predominantly esterified based on ^31^P NMR (0.2 mmol/g free COOH) (Figure 2B). Signals in the HSQC NMR spectrum (Figure 2C) corresponding to -OMe (^1^H 3.69 ppm, ^13^C 55.34 ppm) from methyl ester and ether groups support the presence of galacturonic methyl esters, which is consistent with their endogenous occurrence in plant cell walls. The presence of methyl esters and ethers was further corroborated by hydroiodic acid digestion followed by GC–MS, which determined an -OMe content of 0.7 mmol/g in AIR. However, this technique does not differentiate between methyl esters and ethers and thus also includes contributions from the 4-*O*Me groups expected in arabino-4-*O*-methylglucuronoxylan. The relatively high levels of arabinose (Ara) and galactose (Gal) are likely derived from RG-I pectin side chains, including arabinans, galactans, and type II arabinogalactans. The presence of arabinans in AIR is tentatively assigned from the HSQC signals in the region ^1^H 3.70–3.90 ppm, ^13^C 79.70–84.00 ppm (Figure 2C, Supplementary Table S3). The mannose (Man) content would originate from pectin-associated mannoglycans, proteoglycans, or galactoglucomannans.

##### 3.2.4.2 Xylans

Xylose (Xyl) was detected as both monosaccharide and disaccharide in Driselase digests, including xylobiose (d-Xyl-β-(1→4)-d-Xyl) and isoprimeverose (d-Xyl-α-(1→6)-d-Glc) (Supplementary Figure S1). The additional occurrence of GlcA and 4-*O*-MeGlcA suggests the presence of arabino-4-*O*-methylglucuronoxylan. Its partial extractability in water may be explained by its weak hydrogen bonding ability, which reduces self-aggregation and binding to cellulose. HSQC NMR (Figure 2C, Supplementary Table S3) showed the possible presence of a β-(1→4)-xylan backbone with signals tentatively assigned to xylose and 4-*O*-Me-glucuronic acid groups. Signals tentatively assigned to methyl ethers in 4-*O*-MeGlcA was observed in the HSQC spectra (^1^H 3.37 ppm, ^13^C 58.39 ppm). Additionally, acetylation at C3 of xylose was hypothesized based on the peak at ^1^H 4.94 ppm, ^13^C 73.63 ppm, which may also contribute to its water extractability as a consequence of reduced hydrogen bonding capability leading to reduced association with cellulose fibers.

##### 3.2.4.3 Xyloglucans

Xylose was also detected in the form of isoprimeverose, suggesting the presence of xyloglucans in AIR, albeit in low amounts. Generally, xyloglucans have a β-(1→4)-glucan backbone with a Glu/Xyl ratio ranging from 1.2 to 2, indicating that 70–80% of glucose residues are branched by xylose.^49^ Signals in the HSQC NMR spectrum (Figure 2C, Supplementary Table S3) tentatively assigned to β-(1→4)-glucan backbones and 1,4,6-substituted glucose residues are indicative of the possible presence of xyloglucans. Additional signals tentatively assigned to the α-Xyl*p*-(1→*x*) side group on the glucan backbone were also observed.

##### 3.2.4.4 Other Glucose-Rich Polysaccharides

The high glucose content of AIR observed in total hydrolysis suggests the presence of an additional glucose-rich polysaccharide. The absence of cellobiose in the Driselase digests indicates that cellulose is absent, as expected. Other possible glucans include callose (β-(1→3)-glucan) or mixed-linkage β-(1→3), (1→4)-glucans. Callose, typically found in phloem sieve plates,^50^ may contribute to AIR, although hemicellulosic β-glucans have not yet been reported for WRC.

### 3.3 Proanthocyanidin Composition of AIR

The presence of proanthocyanidins in WRC bark AIR was visually inferred from the formation of red pigments during acidic methanolysis. However, a methylcellulose precipitation assay (Supplementary Methods)—initially employed to verify the presence of tannins—showed only negligible amounts. Furthermore, results from mild acidic methanolysis of AIR suggested the absence of hydrolyzable tannins (Figure S2).

#### 3.3.1 Depolymerization of Proanthocyanidins

Depolymerization assisted by benzylthiol was used to determine the monomers of proanthocyanidins in AIR. Flavan-3-ol benzyl thioethers formed already within 30 min at 40 °C, but extending the reaction to 48 h at room temperature further increased the yield (Figures S3 and S4), particularly for prodelphinidin units. On average, AIR contained 1.46% (w/w) proanthocyanidins, a value comparable to other cell wall preparations, such as ferulic acid in spinach cell walls (0.5% w/w)^39^ and maize cell walls (0.5–1.8% w/w).^51^ However, the proanthocyanidin content of AIR is likely underestimated due to the presence of polysaccharides, which are competitors to the sulfur reagents nucleophilic attack on the C4 position of proanthocyanidins.^52^ Note that 4-*O*-MeGlcA moieties, after acid-catalyzed methanol elimination, would give rise to highly reactive intermediates of the hexenuronic acid type, which, for instance, would consume the thiol reagent.

#### 3.3.2 Insights from ^31^P NMR

The ^31^P NMR analysis of phosphitylated AIR (Figure 2B) showed 0.6 mmol/g of phenolic OH groups (136.50–144.25 ppm)—three times the amount estimated by thiolysis (0.2 mmol/g). The higher phenolic OH content observed in ^31^P NMR suggests that approximately two-thirds of the detected polyphenols were not cleaved or identified by thiolysis. Bonds resistant to thiolytic cleavage, such as ether-linked C7–O–C2 (A-type proanthocyanidins) and C4–C6 linkages (branched proanthocyanidins), as well as the mentioned side reactions, account for this discrepancy.

#### 3.3.3 Proanthocyanidin Structure

Thiolysis of AIR revealed four types of terminal units: gallocatechin, epigallocatechin, catechin, and epicatechin, in a molar ratio of 0.9:0.1:1.0:2.9. Four extension units were also detected, consisting of procyanidin (catechin and epicatechin) and prodelphinidin (gallocatechin and epigallocatechin) types. While the earlier study by Hergert^9^ reported only procyanidins in WRC bark, this study identifies prodelphinidin units as well. On average, AIR proanthocyanidins exhibited a *cis*/*trans* ratio of 0.40, with a procyanidin-to-prodelphinidin (PC/PD) ratio of 3.9, and were composed predominantly of catechin (65% mol).

The absence of methyl gallate in mild acidic methanolysis (Supplementary Figure S2) suggests a lack of galloylation in the AIR proanthocyanidins. Signals assigned to aromatic ring B protons of procyanidins (^1^H 6.65, 6.94, and 7.15 ppm, ^13^C 105 ppm) were observed in the ^1^H and HSQC NMR spectra of AIR (Figure 2A,C). However, the absence of additional characteristic proanthocyanidin peaks prevented further structural insights.

The thiolysis results indicate that AIR contains both procyanidin and prodelphinidin units with a predominance of catechin-based procyanidins. A substantial portion of proanthocyanidins in AIR remains resistant to depolymerization, likely due to the presence of A-type linkages and strong associations or side reactions with polysaccharides. These findings underscore the complexity of AIR’s polyphenol composition and its interactions with cell wall components, which impact its extractability and reactivity.

### 3.4 Noncovalent Associations of the Components of AIR

A pivotal observation in this study was the drastic change in the water solubility of AIR isolated from WRC bark fibers after drying. While the dried aqueous extract redissolved readily in water, AIR displayed poor water solubility after drying. This phenomenon, referred to as “hornification,” occurs when polysaccharides lose water solubility due to expulsion of solvent molecules keeping the chains apart, the collapse of interchain distances and aggregation of their polymer chains during drying.^53^ In the aqueous extract, hornification is avoided due to the presence of the solvent and methanol- or acetone-soluble components that act as spacers, preventing the polysaccharide chains from aggregating and forming dense, insoluble agglomerates during drying.^53,54^

Proanthocyanidins in WRC bark and AIR are strong candidates for such spacer components. Their presence was initially inferred from the red pigments formed during digestion in hot alcoholic acid^55^ and later confirmed by thiolysis. Polyphenols, such as proanthocyanidins, are known to significantly influence the water-holding and -swelling capacities of fiber systems. However, their effects can vary: while some polyphenols improve these properties,^56^ others can diminish them,^57^ depending on the specific polysaccharide-polyphenol combination. For example, catechin, a type of flavanol, has been shown to enhance water-holding capacity and improve swelling in polysaccharide systems.^58^

In this study, the removal of flavanol monomers and polymers in the aqueous extract likely reduced the availability of polyphenolic groups capable of interacting with polysaccharides. This decrease would impair water uptake kinetics and result in decreased swelling, dispersibility, and solubility of AIR. A similar phenomenon was observed in spruce bark aqueous extracts by Bishop^59^ and Harpham,^45^ who reported that the removal of tannins through dialysis led to the precipitation of some polysaccharide components. These findings suggest that polyphenols in the extract serve a crucial role in maintaining the solubility and stability of polysaccharide systems through noncovalent interactions.

To test whether the hornification effect in WRC bark AIR could be reversed, a 0.1% (w/v) catechin solution was used as a dispersant. However, AIR did not disperse, and the results were comparable to those with water alone. This finding suggests that the acetone- and methanol-soluble components present in the aqueous extract are critical for preventing hornification by acting as polysaccharide spacers. Their removal likely contributed to the poor solubility and dispersibility of AIR after drying.

Proanthocyanidins that remain in the system after washing with neutral organic solvents are hypothesized to contribute to the hydration and swelling of polysaccharides, even though they do not lead to complete dissolution. This behavior is comparable to that of common insoluble dietary fibers such as locust bean gum and feruloylated arabinoxylans. While not fully water-soluble, these materials exhibit significant water-holding capacity due to the presence of hydrophilic moieties capable of retaining water within their structure.^60^ Additionally, physical interactions between polysaccharide chains and phenolic moieties can promote gelation and influence gel properties, as demonstrated in the case of feruloylated arabinoxylans.^61^ Similar characteristics may be expected for the WRC bark AIR obtained in this study, which consists primarily of polysaccharides associated with proanthocyanidins. These findings merit further investigation. Given the mild conditions used to obtain this extract, the resulting molecules are well-suited for potential high-value applications in the food and nutraceutical sectors.

#### 3.4.1 Observations on Solubility and Dispersibility in Various Solvent Systems

In an effort to separate the polysaccharide and polyphenolic components of AIR, various solvent systems commonly used for solubilizing cell wall materials^62,63^ or insoluble proanthocyanidin-protein complexes^64^ were tested at room temperature. The results, summarized in Supplementary Table S4, reveal insights into the noncovalent interactions among the components of AIR. The color of the supernatant and residue was taken as an indirect indication of the solubility of proanthocyanidin (yellow to slightly brown) and its associated components. Despite testing chaotropic agents, chelating agents, alkali, and reagents disrupting hydrophobic interactions, none of the solvents achieved complete dissolution of AIR, as seen by centrifugation. Instead, these solvents showed varying degrees of dispersion, providing clues about the interactions among the AIR components.

#### 3.4.2 Buffered Water and Dimethyl Sulfoxide

Water (buffered and unbuffered) and dimethyl sulfoxide (DMSO), usually effective for starch and hemicelluloses that are heavily acetylated, provided poor dispersions, indicating strong associations between AIR components.

#### 3.4.3 Chaotropic Reagents

Chaotropic agents, such as urea and guanidinium thiocyanate, produced swollen dispersions, suggesting strong hydrogen bonding within AIR. Urea dispersion resulted in a nitrogen content increase of +0.5% in AIR, indicating possible incorporation or very strong adsorption. However, the brown residue indicated that some proanthocyanidins remained associated.

#### 3.4.4 Chelating Agents

Chelating agents, such as ethylenediaminetetraacetic acid (EDTA) and *trans*-1,2-cyclohexanediaminetetraacetic acid (CDTA) produced poor dispersions, suggesting minimal contributions of calcium ion bridges commonly observed in pectin or also alginates. Interestingly, imidazolium hydrochloride (a dual chaotropic and calcium-complexing agent) dispersed AIR (turbid suspension), but the addition of EDTA to this system prevented dispersion.

#### 3.4.5 Alkaline Systems

Alkaline reagents provided the least turbid dispersions and produced the lightest colored residues. Hydroxide ions, known for their chaotropic effect and ability to cleave alkali-labile bonds (e.g., esters), likely contributed to the solubilization of polysaccharides. Basic conditions also depolymerize proanthocyanidins,^29^ making it unlikely that these dispersions were achieved without degradation of AIR components. NaBH_4_ in alkaline media, often used to minimize polysaccharide peeling reactions, can also cleave proanthocyanidins by mild alkaline reductive cleavage.^65^ Higher alkali concentrations decrease hydrolysis of NaBH_4_ ^66^ and the amount of generated H_2_ so that the larger amount of hydride donors present can better cleave the interflavonoid bond in proanthocyanidins; however, the extent of depolymerization under these conditions requires further investigation.

#### 3.4.6 Hydrophobic Interaction Disruptors

Reagents that disrupt proanthocyanidin complexation^67^ used to target proanthocyanidin hydrophobic interactions, such as poly­(ethylene glycol) (PEG, 4 kDa) and sodium dodecyl sulfate, failed to dissolve AIR (depicted as the proposed structure in Figure 4). The brown residues left after centrifugation indicated that proanthocyanidins remained largely associated with polysaccharides.

**Figure 3:**
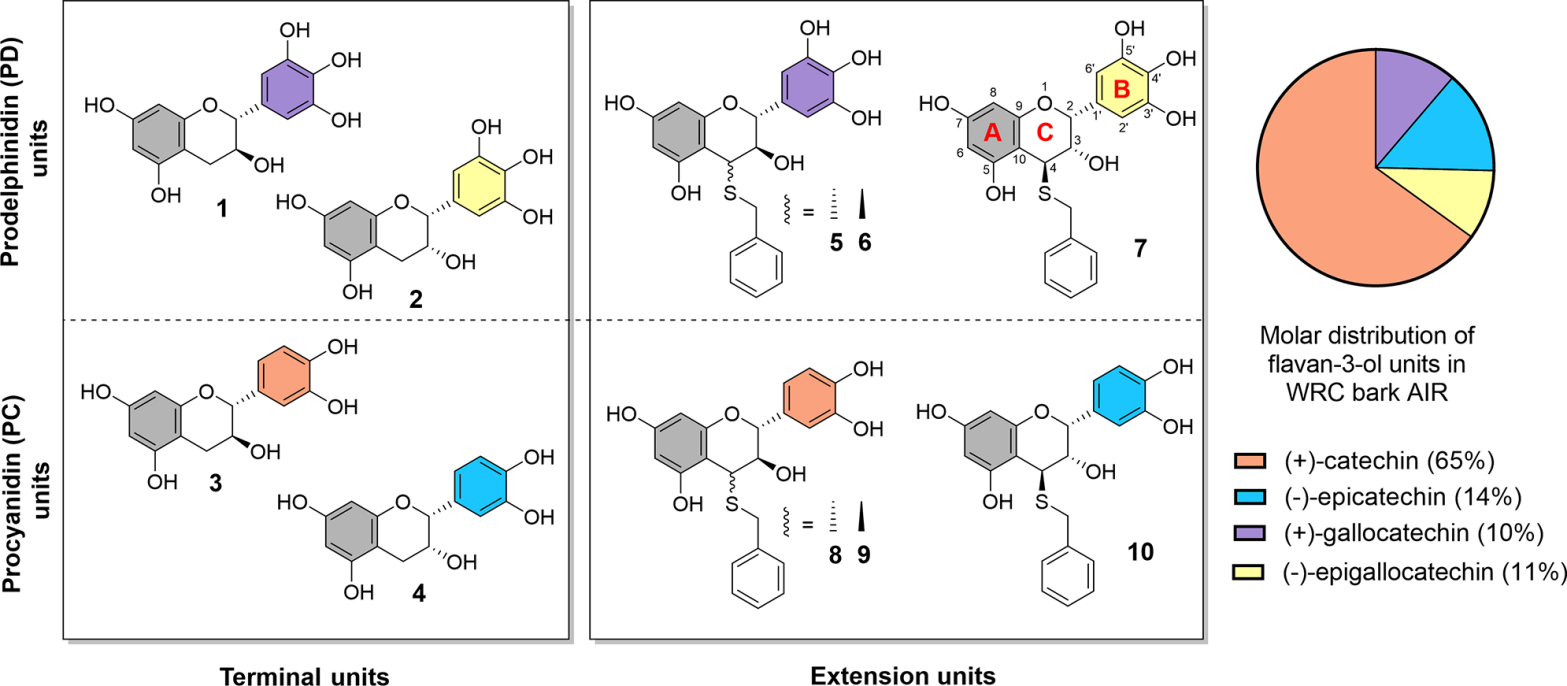
Proanthocyanidin monomer subunits (% mol) in AIR detected after thiolysis quantified by HPLC–UV.

**Figure 4:**
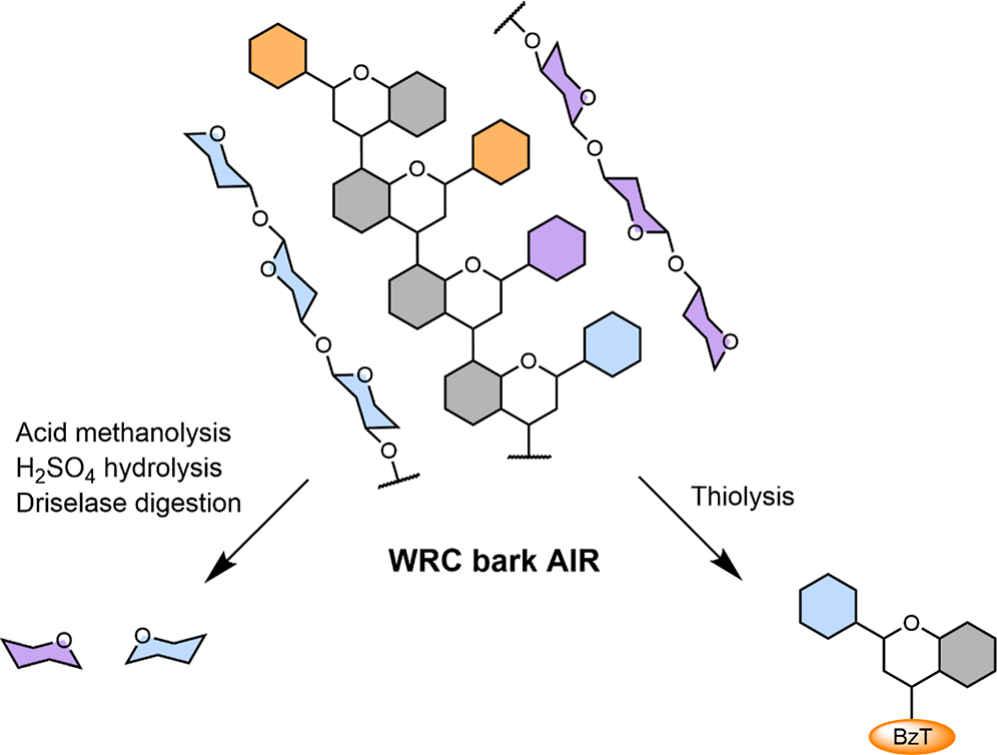
Graphical representation of proanthocyanidins and polysaccharides in AIR and the cleavage products detected using different depolymerization methods. BzT = benzyl thioether.

### 3.5 Polysaccharide–Polyphenol Interactions Result in Strong Association

The obtained results show that aqueous extracts of WRC bark contained proanthocyanidins and polysaccharides. We hypothesize that this is likely due to the association of proanthocyanidins with pectin, xyloglucans, xylans, and starch. This can be explained by multiple structural and chemical features that we have observed, most of which have already been studied in laboratory model systems to play a role in proanthocyanidin-polysaccharide interactions.

#### 3.5.1 Methyl Esterification of Pectin and Rhamnose Content

Methyl esterification of uronic acids in pectin enhances hydrophobicity and induces an extended conformation, facilitating association with proanthocyanidins.^68^ In addition, the rhamnose residues present in HG and RG-I chains increase the flexibility of pectin and can improve association with proanthocyanidins.

#### 3.5.2 Xyloglucans and Xylans

The substituted glucan backbone of xyloglucans forms semiflexible coils capable of hydrophobic interactions with proanthocyanidins. While the binding of proanthocyanidins to xylans has not yet been reported, their presence in WRC bark AIR suggests that xylans may contribute to the observed associations between AIR polysaccharides and proanthocyanidins. Xylose residues, which provide a relatively hydrophobic surface,^69^ may play a role in proanthocyanidin association, although their specific contribution requires further study. The acetylation of the xylan chain in AIR, tentatively assigned in HSQC NMR (Figure 3C and Supplementary Table S3), provides additional hydrophobic surfaces that are likely to contribute to the interactions.

#### 3.5.3 Catechin Units in Proanthocyanidins

The abundance of catechin units in proanthocyanidins imparts an open and flexible conformation, enhancing their ability to associate with pectins and hemicelluloses like xyloglucan.^20,70^

Overall, the inability of various solvent systems to fully dissolve AIR highlights the complexity and strength of the noncovalent associations among its components. The coextraction of proanthocyanidins with polysaccharides, such as pectin, xyloglucans, and xylans, suggests a significant role for hydrophobic interactions, enhanced by structural features like methyl esterification, acetylation, and the flexible conformation of catechin-rich proanthocyanidins. These associations emphasize the unique properties of AIR and provide avenues for further study of the mechanisms driving these interactions.

### 3.6 Implications of Proanthocyanidin–Polysaccharide Interactions in WRC Bark

The co-occurrence of proanthocyanidins with polysaccharides in AIR derived from WRC bark fibers supports and agrees with the previously proposed model of noncovalent binding between proanthocyanidins and cell wall polysaccharides.^20,68^ In this study, we observed that the binding of initially soluble proanthocyanidins to polysaccharides is remarkably robust and persistent also in the presence of other compounds, e.g., those reported to occur in crude bark extracts, such as monomeric flavonoids, phenolic acids, aldehydes, fats, and waxes,^3–8^ and resisting disruption by organic solvents known to dissolve proanthocyanidins.

Proanthocyanidins and melanins are known to be concentrated in highly resistant plant tissues like bark,^71^ where they play vital physiological roles in protecting plants from biotic and abiotic stresses.^72^ Upon cellular damage, polyphenolic parenchyma cells burst, releasing proanthocyanidins, which deposit on the cell walls of necrotic and adjacent cells. WRC has been reported to develop injury-induced decay resistance,^73^ hypothesized to result from the deposition of a fungicide strongly associated with bark fibers. While previous studies have not successfully isolated or identified the specific components responsible for this behavior, we hypothesize, through the findings of this study, the formation of proanthocyanidin-polysaccharide complexes. These complexes, observed to be difficult to disrupt, separate, or depolymerize, likely contribute to WRC’s decay resistance. Such structures are known for their antimicrobial properties and reduced digestibility,^74^ meeting their proposed role in protecting injured WRC bark. The cooperative effects of various noncovalent interactions within AIR, including hydrogen bonding and hydrophobic interactions, may explain the stability and resilience of these complexes.

The possibility of covalent bonds between proanthocyanidins and polysaccharides, although not explored in detail here, remains an intriguing topic for future investigation. We will address this in a subsequent report. The current study represents the first detailed structural characterization of proanthocyanidins and polysaccharides in WRC bark AIR with a focus on their noncovalent interactions. These findings provide a foundation for rethinking the valorization of this material, emphasizing the potential to harness the unique properties of these strongly associated structures.

## 4 Conclusions

Aqueous extraction of WRC bark offers a valuable approach for industrial utilization, yielding both polysaccharides and proanthocyanidins with significant commercial potential. This study demonstrated that polysaccharides (pectins, xyloglucans, and xylans) and catechin-based procyanidins extracted from WRC bark exhibit structures conducive to their strong association. As a consequence, the separation of proanthocyanidins from polysaccharides proved challenging, as their interactions are stabilized by a combination of noncovalent interactions. These interactions may contribute to the natural resistance mechanisms observed in the WRC, highlighting the importance of these complexes. Future valorization efforts should account for the presence of these strongly associated structures to enable more economical extraction processes and uncover functional properties for commercial applications.

## Supplementary Material



## Notes

Additional experimental details, materials and methods, and information on the recoveries of different polysaccharide methods, summary of assignments and integration of NMR spectra, results of dispersibility of AIR in various solvent systems, and chromatograms of polysaccharide and proanthocyanidin analyses (PDF)

## Abbreviations

WRC: western redcedar
AIR: alcohol-insoluble residue
NMR: nuclear magnetic resonance spectroscopy
HPLC-DAD-MS: high-performance liquid chromatography-diode array detection-mass spectrometry
mDP: mean degree of polymerization
HPAEC-PAD: high-performance anion-exchange chromatography-pulsed amperometric detection
GC-MS: gas chromatography-mass spectrometry
HSQC: heteronuclear single quantum coherence
GARP: globally optimized alternating-phase rectangular pulses
Glu: glucose
Ara: arabinose
Gal: galactose
Xyl: xylose
Man: mannose
Rha: rhamnose
Fuc: fucose
GalA: galacturonic acid
4-*O*-MeGlcA: 4-*O*-methylglucuronic acid
GlcA: glucuronic acid
HG: homogalacturonan
RG I: rhamnogalacturonan I
