# Supplementary material for: Strong Association between Proanthocyanidins and Polysaccharides in the Cell Walls of Western Redcedar Bark
Source: Biomacromolecules. 2025 May 6;26(9):5601–13. doi: 10.1021/acs.biomac.5c00271 (PMC12421503; doi:10.1021/acs.biomac.5c00271)
Supplement: Supplementary file 1 [file bm5c00271_si_001.pdf]

Supporting Information

Strong association between proanthocyanidins and polysaccharides in the cell walls of western redcedar bark

Gio Ferson M. Bautista[a,b], Oliver Musl[b,c,*], Michael L. A. E. Easson[d], Lars H. Kruse[d], Harley Gordon[e], Markus Bacher[c], Ivan Sumerskii[f], Aude A. Watrelot[g], Jörg Bohlmann[d,h,i], Antje Potthast[c], Thomas Rosenau[c], Orlando J. Rojas[a,b,j,k,l,*]

[a] Department of Chemistry, The University of British Columbia, 2036 Main Mall, Vancouver, BC V6T 1Z1, Canada

[b] Bioproducts Institute, The University of British Columbia, 2385 East Mall, Vancouver, BC V6T 1Z4, Canada.

[c] Department of Natural Sciences and Sustainable Resources, Institute of Chemistry of Renewable Resources, University of Natural Resources and Life Science (BOKU), Vienna, Tulln A-3430, Austria

[d] Michael Smith Laboratories, The University of British Columbia, Vancouver, BC, Canada

[e] Faculty of Land and Food Systems, The University of British Columbia, 2357 Main Mall, Vancouver, BC V6T 1Z4, Canada

[f] Core Facility "Analysis of Lignocellulosics" (ALICE), University of Natural Resources and Life Sciences, Vienna, Tulln A-3430, Austria

[g] Department of Food Science and Human Nutrition, Iowa State University, 536 Farm House Lane, Ames, IA 50011, USA

[h] Department of Botany, The University of British Columbia, 6270 University Blvd., Vancouver, BC V6T 1Z4, Canada

[i] Department of Forest and Conservation Sciences, The University of British Columbia, 2424 Main Mall, Vancouver, BC V6T 1Z4, Canada

[j] Department of Chemical and Biological Engineering, University of British Columbia, 2360 East Mall, Vancouver, BC V6T 1Z3, Canada.

[k] Department of Wood Science, The University of British Columbia, 2424 Main Mall #2900, Vancouver, BC V6T 1Z1, Canada

[l] Department of Bioproducts and Biosystems, Vuorimiehentie 1, Aalto University, Espoo, FI-00076 Finland

*Corresponding authors: oliver.musl@boku.ac.at, orlando.rojas@ubc.ca, orlando.rojas@aalto.fi

Supplementary Methods

Determination of starch content by amylolytic enzyme digestion. The starch content of the alcohol-insoluble residue (AIR) was determined by enzymatic digestion as reported by Theander and Westerlund[1], followed by quantification of glucose by HPAEC-PAD. About 100 mg of AIR was suspended in 10 mL of 100 mM acetate buffer pH 5. 50 µL of Termamyl 120 (20 mg protein/mL, 834 enzyme units) was added for enzymatic digestion. The mixture was tightly capped and was kept in an oil bath at 100 °C for 30 min with occasional shaking. After cooling down to room temperature, 1 mL of amyloglucosidase (1 mg/mL, 142 enzyme units) was added. The mixture was tightly capped and was left at 60 °C overnight (at least 16 h). The mixture was then cooled and filtered through a crucible and the supernatant was analyzed for glucose content by HPAEC-PAD. Native starch from wheat was used as positive control, while β-glucan from barley (high viscosity) and Avicel PH-101 were used as negative controls. Values reported were corrected for sugar content of the enzyme mixtures.

Determination of neutral monosaccharides, disaccharides, and uronic acids by HPAEC-PAD. HPAEC-PAD analysis was performed on samples from total hydrolyses in H_2SO_4, amylolytic enzyme digests, and Driselase digests. A Dionex ICS-6000 HPIC (Thermo Fisher Scientific, MA, USA) equipped with a Dionex CarboPac PA1 column and a Dionex CarboPac PA1 guard column, AXP auxiliary pump, Dionex AS-AP autosampler, and a Pulsed Amperometric Detector with a gold electrode on a PTFE surface was used in the analysis. The default quadruple-potential waveform [2] was used for detection, and the signal was recorded as the resulting current (in nA) during the detector integration period. Before injection, 950 µL of the sample (or standard) was mixed with 50 µL of internal standard (D-sorbitol) and filtered through a 0.45 µm PTFE syringe filter. Water was used as Solvent A and 1 M NaOH in water as Solvent B. For the analysis of monosaccharides and uronic acids, 12.5-µL aliquot of the sample was injected onto the column and eluted at 0.4 mL/min using the solvent program as follows: 0-5.0 min 0.5% B, 5.0-5.1 min, decrease to 0% B,

5.1-85 min 0% B, 85.0-125.0 min increase to 80% B, 125.0-135.0 min 80% B, 135.0-135.1 min decrease to 0.5% B, 135.0-145.0 min 0.5% B. 200 mM NaOH was added post-column at a flow rate of 0.5 mL/min for the detection.

For disaccharide analysis, 12.5-μL aliquot of the sample was injected into the same system and eluted at 0.4 mL/min using a different solvent program as follows: 0-10.0 min 5% B, 10.0-30.0 min 5% B, 30.0-30.1 min increase to 80% B, 30.1-35.0 min 80% B, 35.0-35.1 min decrease to 5% B, 35.1-45.0 min 5.0% B.

For glucose analysis after amylolytic enzyme digestion, 10.0-μL aliquot of the sample was injected into the same system and eluted at 1.0 mL/min flow rate using a different solvent program as follows: 0-35.0 min 0% B, 35.0-40.0 min 25% B, 40.0-50.0 min 0% B.

Determination of Monosaccharides by GC-MS. Samples for Gas Chromatography-Mass Spectrometry (GC-MS) were from methanolysis of samples. The analysis was performed with an Agilent 6890N GC and an Agilent 5975 Binert XL MSD quadrupole mass-selective detector (EI: 70 eV), by using a non-polar Agilent HP-5MS capillary column (30 m × 0.25 mm i.d.; 0.25 mm film thickness), and helium as the carrier gas with a pressure of 0.94 bar and a flowrate of 1.1 mL/min. The temperature program was as follows: hold at 50°C for 2 min, ramp to 280°C @5°C/min, hold at 280°C for 20 min.

Determination of Methoxyl Group Content by HI Digestion Followed by Headspace GC. Methoxyl groups in WRC bark AIR were quantified following Sumerskii et al.[3]. Briefly, 10 mg of sample and 3 mg of internal standards (4-(methoxy-d_3)-benzoic acid and 4-(ethoxy-d_5)-benzoic acid) were added with 1 mL of HI acid (57% w/w) in a 10 mL screw-cap headspace vial. The

mixture was magnetically stirred while heating at 110 °C for 3 h. After cooling to room temperature, 4 mL of water was added prior to Headspace GC-MS analysis[3].

Quantification of Tannins Using the Methylcellulose Precipitation Assay. The tannins in WRC bark AIR were quantified using UV-Vis spectrophotometry by the methylcellulose precipitation assay[4]. Briefly, 10 mg of the extract was dispersed in 1 mL of 50% (v/v) aqueous ethanol. The mixture was then centrifuged at 13,000 × *g* for 10 min and the supernatant was taken for spectrophotometric analysis. In the analysis, 100 µL of the sample was mixed with 300 µL of 0.04% (w/v) of methylcellulose and 200 µL of saturated ammonium sulfate solution, and 400 µL of water. The mixture was mixed in a vortex mixer and then was allowed to stand for 10 min at room temperature. The mixture was then centrifuged at 10,000 rpm for 5 min and the supernatant was used and the absorbance at 280 nm was recorded. For the control, 100 µL of the sample was added with 200 µL of saturated ammonium sulfate solution and 700 uL of water. The mixture was mixed in a vortex mixer, and then was allowed to stand for 10 min at room temperature. The mixture was then centrifuged at 10,000 rpm for 5 min, and the supernatant was used and the absorbance at 280 nm was recorded.

Determination of Hydrolysable Tannins by Mild Acid Methanolysis. WRC bark AIR was subjected to mild acid methanolysis for the detection of gallic acid, tannic acid, ellagic acid, and their derivatives, the common hydrolysable tannins in wood and bark. The procedure by Watrelot et al.[5] was followed. Briefly, 3 mg of sample was dispersed in 2.5 mL of 0.6 N HCl in methanol. The mixture was heated at 120 ºC for 4 h, and the reaction was stopped by cooling in an ice bath. The sample was centrifuged at 17000 × *g* for 1 minute, and 0.50 mL of the supernatant was diluted to 1 mL using water in a volumetric flask. The diluted sample was filtered through a 0.45 µm PTFE filter prior to LC-UV analysis, using the same HPLC run parameters as for thiolysis.

Determination of Thiolysis Products by HPLC-DAD and LC-MS. Controls and products of thiolysis were analyzed in an Agilent HPLC 1200 equipped with a diode array detector and a fluorescence detector. The column used was a Poroshell HPH-C18 2.7 µm (4.6 x 100 mm). A flow rate of 0.6 mL/min at 30 °C was used with Solvent A as 0.13% (v/v) trifluoroacetic acid (TFA) in water and Solvent B as 0.10% (v/v) TFA in acetonitrile. The solvent programming was as follows: 0.0-1.5 min 15% B, 1.5-4.0 min increase to 20% B, 4.0-5.0 min increase to 30% B, 5.0-14.0 min 30% B, 14.0-16.0 min increase to 70% B, 16.0-18.0 min 70% B, 18.0-20.0 min increase to 90% B, 20.0-24.0 min 90% B, 24.0-24.5 min decrease to 15% B, 24.5-27.0 min 15% B. The sample mixture was filtered through a 0.45 µm PTFE filter prior to the injection of 5 µL of the sample. The absorbance of the eluate at 280 nm was monitored and the integrated areas in the chromatogram at this wavelength were used for quantification.

For the ESI-Ion Trap-MS^n analysis, a Hewlett Packard HPLC 1100 equipped with a diode array detector and a Bruker HCT Ultra PTM Discovery System was used. The column used was Atlantis T3 3 µm (3.0 x 100 mm) with a guard column (4.6 x 20 mm) of the same material. A flow rate of 0.6 mL/min at 30 °C was used with Solvent A as 0.13% (v/v) formic acid in water and Solvent B as 0.10% (v/v) formic acid in acetonitrile. The solvent programming was as follows: 0.0-4.5 min 15% B, 4.5-7.0 min increase to 20% B, 7.0-8.0 min increase to 30% B, 8.0-19.0 min 30% B, 19.0-21.0 min increase to 70% B, 21.0-26.0 min 70% B, 26.0-28.0 min increase to 90% B, 28.0-35.0 min 90% B, 35.0-35.5 min decrease to 15% B, 35.5-38.0 min 15% B. The sample mixture was filtered through a 0.45 µm PTFE filter prior to injection of 5 µL of the sample. The absorbance of the eluate at 190-950 nm was monitored. The ESI-MS analysis was done in negative ion mode at an m/z range of 50-1050 m/z, and an average of 5 spectra were collected for each data point. In negative ion mode, capillary voltage of +3500 V, nebulizer pressure of 50 psi, and dry gas flow rate of 10 L/min at 350 °C were used. The Smart Setting of the software was given a target mass

of 500 m/z, a compound stability of 50%, and a trap drive level of 90%. At these settings, the following were automatically set by the software: skimmer of -40.0 V, capillary exit of -110.7 V, Oct 1 DC of -8 V, Oct 2 DC of 1.7 V, Octopole RF of 187.1 Vpp, Lens 1 of -5 V, Lens 2 of -60 V, and a trap drive of 53.9. ICC was activated and manually set to a value of 70,000 and a maximum accumulation time of 100 ms. For MS^n fragmentations in the negative ion mode, an isolation width of 4 m/z was used, and the fragmentation amplitude was adjusted from 0.5-2.0 V.

Qualitative Determination of Dispersibility of AIR in Various Solvents. A total of 1 mg of AIR was added to 1 mL of test solvent and was mixed by a magnetic stirrer for 24 hours. Afterwards, the relative turbidity of the sample was recorded relative to other samples. The mixture was centrifuged at 17,000 × *g* for 5 min at room temperature and the color of the supernatant and the pellet was recorded.

Supplementary Tables

Table S1. %Recovery in WRC bark AIR for different polysaccharide methods (dried sample basis)

	Total hydrolysis	Acidic methanolysis	Driselase
%Monosaccharide recovery, w/w	20 ± 2	21.9 ± 0.7	11.3 ± 0.9
%Insoluble residue, w/w	88 ± 11	48	67 ± 5
%Total, w/w	108	70	78

Table S2. Integration and peak assignments for ^{31}P NMR of phosphitylated WRC bark AIR.

Chemical shift, ppm	Assignment	Amount, mmol/g AIR
151.30 – 152.40	Internal standard	-
146.10 – 150.00	Aliphatic OH from polysaccharides	1.7
144.45 – 146.00	Aliphatic OH from proanthocyanidins (Ring C OH)	0.4
143.00 – 144.25	Pyrogallol 4’-OH (Ring B)	0.07
138.40 – 142.50	Catechol or vicinal OH in pyrogallol (Ring B)	0.4
136.50 – 138.30	Phloroglucinol OH (Ring A)	0.08
133.50 – 135.50	Uronic acids from polysaccharides	0.2

Table S3. Observed HSQC peaks and their tentative assignments based on assignments lifted from literature.

Assignment	Position	Peak (δ_H, δ_C), ppm
β-1,4-glucans[6]	Glucose	
	CH1	4.31, 102.58
	CH2	3.04, 72.92
	CH3	3.41, 74.45
	CH4	3.37, 78.55
	CH5	3.25, 75.50
	CH6	3.52, 60.85
Substituted β-1,4-glucans (Xyloglucans)[7]	C6-substituted glucose	
	CH1	Overlap with β-1,4-glucans
	CH2	Overlap with β-1,4-glucans
	CH3	Overlap with β-1,4-glucans
	CH4	Overlap with β-1,4-glucans
	CH5	3.50, 72.26
	CH6	3.60, 67.28
		3.54, 67.53
	Xylose	
	CH1	-
	CH2	-
	CH3	3.39, 71.97
	CH4	3.27, 70.09
	CH5	3.31, 61.25
β-1,4-xylans[8]	Xylose	
	CH1	4.84, 101.29 (Reducing end α-Xyl)
	CH2	-
	CH3	3.68, 69.52 (Reducing end α-Xyl)
		3.35, 74.66 (β-1,4-xylan)
		3.40, 72.58 (C2-substituted with 4-*O*MeGlcA)
	CH4	3.65, 77.61 (β-1,4-xylan)
	CH5	3.30, 62.96 (β-1,4-xylan)
		4.05, 63.31 (β-1,4-xylan)
	3-*O*-acetylxylose	
	CH3	4.94, 73.63
	4-OMeGlcA	
	CH4	4-*O*Me: 3.37, 58.39
α-1,5-arabinans[9,10]	CH1	4.70, 107.86
	CH2	3.71, 83.05
	CH3	3.81, 80.58
	CH4	3.85, 82.18
	CH5	3.50, 69.49

→2)-α-Rha*p*-(1→	CH1	-
(Pectin)[10]	CH2	3.86, 77.51
	CH3	3.81, 69.88
	CH4	3.63, 71.05
	CH5	3.73, 69.88
	CH6	-

Table S4. Relative dispersibility of WRC bark AIR in various solvent systems.

Solvent[a]	Dispersibility[b]	Residue	Supernatant
H_2O	-	brown	pale yellow
0.1 mg/mL catechin solution	-	brown	pale yellow
1:1:23 (v/v/v) pyridine:HOAc:H_2O	-	brown	pale yellow
4:1 DMSO:pyridine (v/v)	-	brown	pale yellow
8 M urea, 50 mM sodium acetate pH 5.5	+	brown	brown
6 M guanidinium thiocyanate	+	brown	brown
0.2 M EDTA pH 6.5	-	brown	light brown
0.05 M CDTA, adjust pH to 7.5	-	brown	brown
0.2 M imidazole-HCl, pH 7	+	brown	light brown
0.2 M EDTA, 0.2 M imidazole-HCl, pH 7	-	brown	yellow
0.1 M NaOH	+++	light gray	yellow
0.1 M NaOH, 0.1% (w/v) $NaBH_4$	+++	light gray	yellow
6 M NaOH, 1% (w/v) $NaBH_4$	+++	faint brown	brown
0.05 M NaOH, 0.005 M EDTA	+++	light gray	brown
50 mg/mL PEG 4000 g/mol	-	brown	pale yellow
1% w/v SDS, 5% v/v TEA	-	brown	pale yellow

[a]HOAc is acetic acid, EDTA is ethylenediaminetetraacetic acid, CDTA is *trans*-1,2-cyclohexanediaminetetraacetic acid, PEG is poly(ethylene glycol), and DMSO and dimethylsulfoxide.

[b]Dispersibility: +++ slight turbidity, + turbid, - very cloudy.

Supplementary Figures

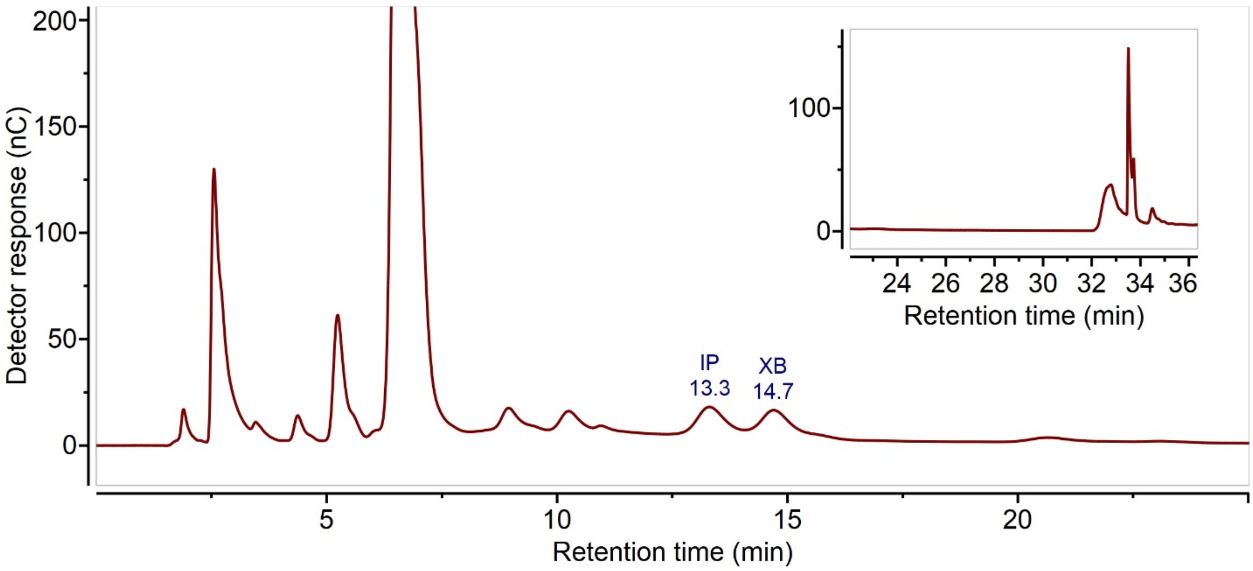

Figure S1. Detection of disaccharides after Driselase digestion. Identity of disaccharides was confirmed by spiking with analytical standards, IP-isoprimeverose, XB-xylobiose. Inset shows the highly retained components (acidic components).

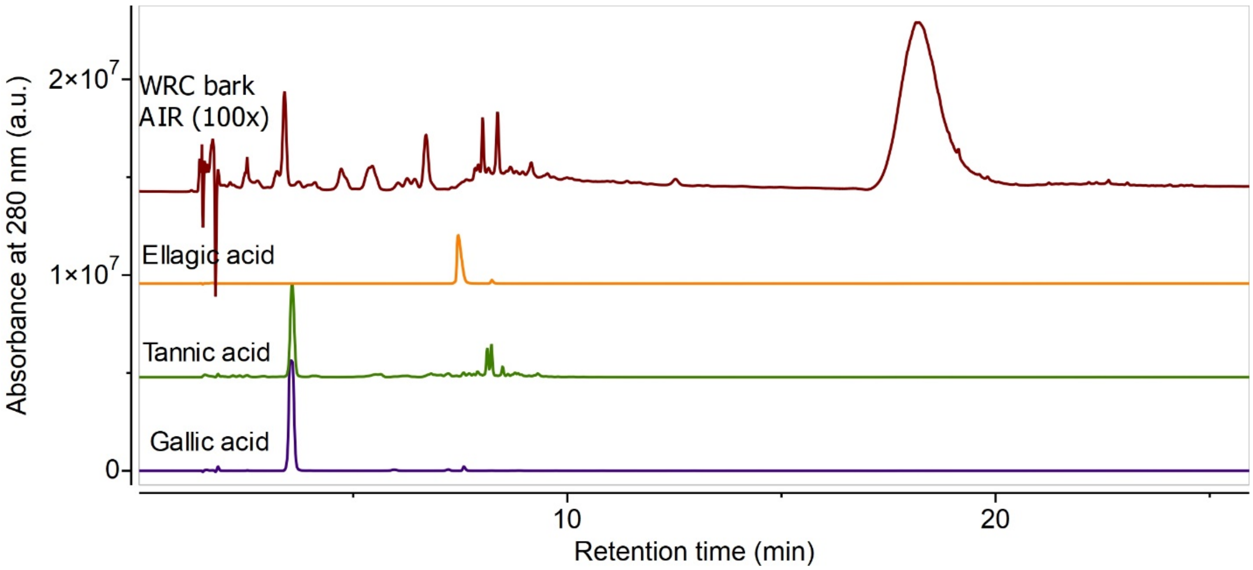

Figure S2. Mild acid methanolysis of WRC bark AIR. Chromatographic trace for WRC bark AIR was multiplied by 100x relative to the others.

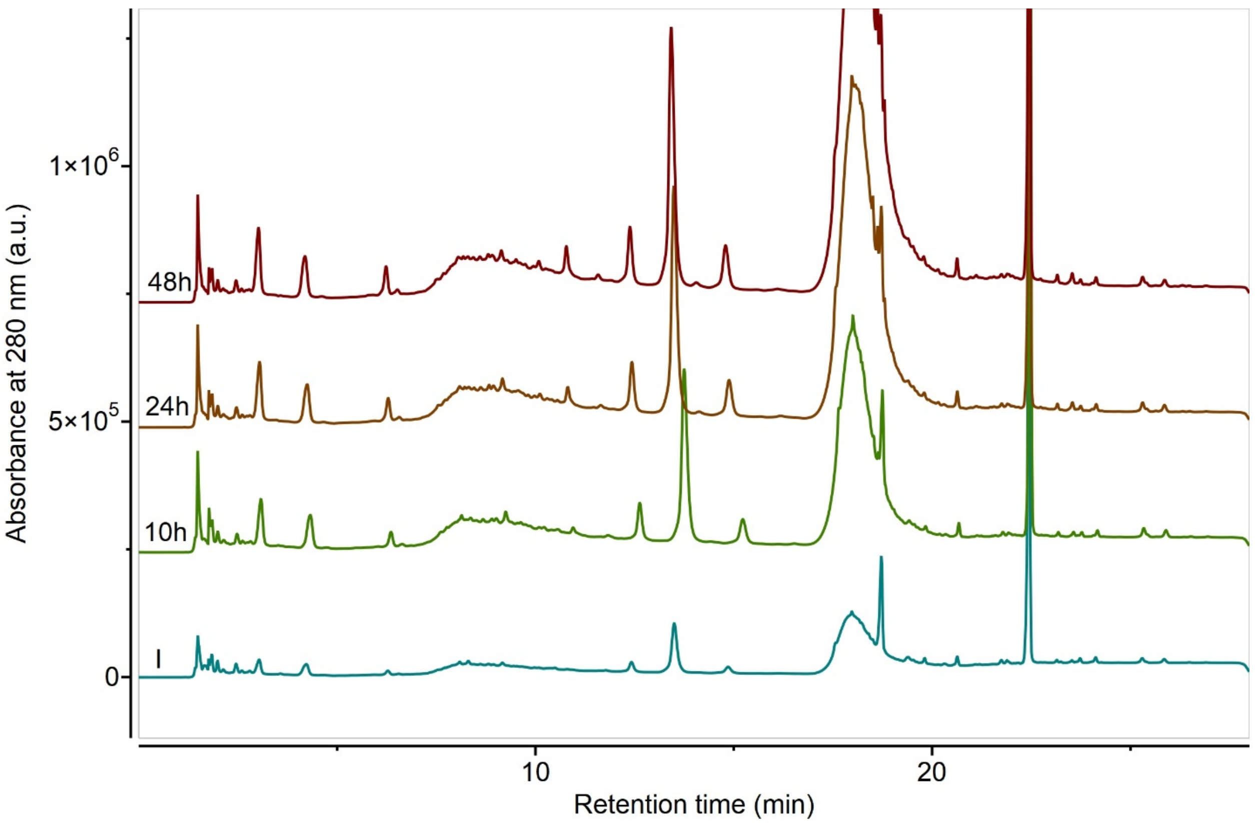

Figure S3. Temperature and time optimization of thiolysis of WRC bark AIR.

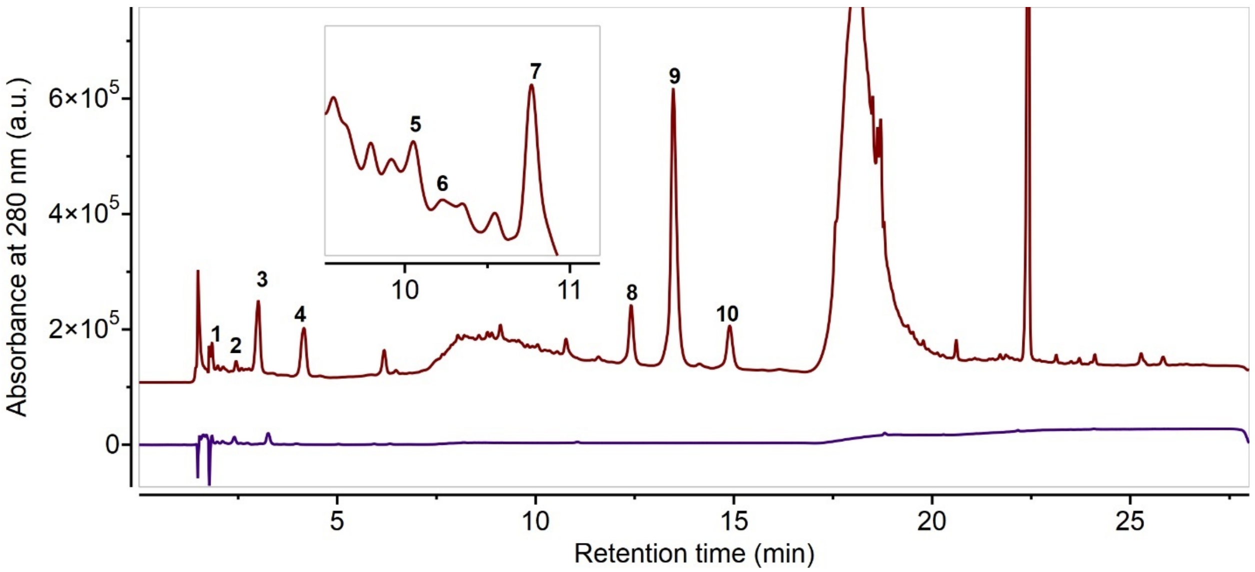

Figure S4. Thiolysis of AIR at the optimum conditions (40 °C for 30 minutes, then 48 hours at room temperature). Peak assignments are shown in Table 2.

References

(1) Theander, O.; Westerlund, E. A. Studies on Dietary Fiber. 3. Improved Procedures for Analysis of Dietary Fiber. *J. Agric. Food Chem.* **1986**, *34* (2), 330–336. DOI: 10.1021/jf00068a045.

(2) Rocklin, R. D.; Clarke, A. P.; Weitzhandler, M. Improved Long-Term Reproducibility for Pulsed Amperometric Detection of Carbohydrates via a New Quadruple-Potential Waveform. *Anal. Chem.* **1998**, *70* (8), 1496–1501. DOI: 10.1021/ac970906w.

(3) Sumerskii, I.; Zweckmair, T.; Hettegger, H.; Zinovyev, G.; Bacher, M.; Rosenau, T.; Potthast, A. A Fast Track for the Accurate Determination of Methoxyl and Ethoxyl Groups in Lignin. *RSC Adv.* **2017**, *7* (37), 22974–22982. DOI: 10.1039/C7RA00690J.

(4) Sarneckis, C. j.; Dambergs, R. g.; Jones, P.; Mercurio, M.; Herderich, M. j.; Smith, P. a. Quantification of Condensed Tannins by Precipitation with Methyl Cellulose: Development and Validation of an Optimised Tool for Grape and Wine Analysis. *Aust. J. Grape Wine Res.* **2006**, *12* (1), 39–49. DOI: 10.1111/j.1755-0238.2006.tb00042.x.

(5) Watrelot, A. A.; Le Guernevé, C.; Hallé, H.; Meudec, E.; Véran, F.; Williams, P.; Robillard, B.; Garcia, F.; Poncet-Legrand, C.; Cheynier, V. Multimethod Approach for Extensive Characterization of Gallnut Tannin Extracts. *J. Agric. Food Chem.* **2020**, *68* (47), 13426–13438. DOI: 10.1021/acs.jafc.9b08221.

(6) Fliri, L.; Heise, K.; Koso, T.; Todorov, A. R.; del Cerro, D. R.; Hietala, S.; Fiskari, J.; Kilpeläinen, I.; Hummel, M.; King, A. W. T. Solution-State Nuclear Magnetic Resonance Spectroscopy of Crystalline Cellulosic Materials Using a Direct Dissolution Ionic Liquid Electrolyte. *Nat. Protoc.* **2023**, *18* (7), 2084–2123. DOI: 10.1038/s41596-023-00832-9.

(7) Silipo, A.; Larsbrink, J.; Marchetti, R.; Lanzetta, R.; Brumer, H.; Molinaro, A. NMR Spectroscopic Analysis Reveals Extensive Binding Interactions of Complex Xyloglucan Oligosaccharides with the Cellvibrio Japonicus Glycoside Hydrolase Family 31 α-Xylosidase. *Chem. - Eur. J.* **2012**, *18* (42), 13395–13404. DOI: 10.1002/chem.201200488.

(8) Kim, H.; Ralph, J. A Gel-State 2D-NMR Method for Plant Cell Wall Profiling and Analysis: A Model Study with the Amorphous Cellulose and Xylan from Ball-Milled Cotton Linters. *RSC Adv.* **2014**, *4* (15), 7549–7560. DOI: 10.1039/C3RA46338A.

(9) Cordeiro, L. M. C.; de Almeida, C. P.; Iacomini, M. Unusual Linear Polysaccharides: (1→5)-α-l-Arabinan, (1→3)-(1→4)-α-d-Glucan and (1→4)-β-d-Xylan from Pulp of Buriti (*Mauritia Flexuosa*), an Edible Palm Fruit from the Amazon Region. *Food Chem.* **2015**, *173*, 141–146. DOI: 10.1016/j.foodchem.2014.10.020.

(10) Makarova, E. N.; Shakhmatov, E. G. Characterization of Pectin-Xylan-Glucan-Arabinogalactan Proteins Complex from Siberian Fir *Abies Sibirica* Ledeb. *Carbohydr. Polym.* **2021**, *260*, 117825. DOI: 10.1016/j.carbpol.2021.117825.
